# Dietary Intake, Mediterranean and Nordic Diet Adherence in Alzheimer’s Disease and Dementia: A Systematic Review

**DOI:** 10.3390/nu17020336

**Published:** 2025-01-17

**Authors:** Christiana C. Christodoulou, Michalis Pitsillides, Andreas Hadjisavvas, Eleni Zamba-Papanicolaou

**Affiliations:** 1 Neuroepidemiology Department, The Cyprus Institute of Neurology and Genetics, Nicosia 2371, Cyprus; christianachr@cing.ac.cy (C.C.C.); michalisp@cing.ac.cy (M.P.); 2 Cancer Genetics, Therapeutics and Ultrastructural Pathology Department, The Cyprus Institute of Neurology and Genetics, Nicosia 2371, Cyprus

**Keywords:** Alzheimer’s disease, dementia, Mediterranean diet, Nordic diet, cognitive decline, cognitive function, dietary patterns, dietary intake, systematic review

## Abstract

Background/Objectives: Dementia is not a single disease but an umbrella term that encompasses a range of symptoms, such as memory loss and cognitive impairments, which are severe enough to disrupt daily life. One of the most common forms of dementia is Alzheimer’s Disease (AD), a complex neurodegenerative condition influenced by both genetic and environmental factors. Recent research has highlighted diet as a potential modifiable risk factor for AD. Decades of research have explored the role of dietary patterns, including the Mediterranean Diet (MD) and its components, in neuroprotection and cognitive health. Systematic review examines studies investigating the impact of the Mediterranean Diet, Mediterranean-like diets, the Nordic Diet (ND), dietary intake patterns, and specific components such as extra virgin olive oil and rapeseed oil on cognitive function, disease onset, and progression in AD and dementia. Methods: A comprehensive search of PubMed, the Directory of Open Access Journals, and the Social Science Research Network was conducted independently by two reviewers using predefined search terms. The search period included studies from 2006 to 2024. Eligible studies meeting the inclusion criteria were systematically reviewed, yielding 88 studies: 85 focused on the MD and its relationship to AD and dementia, while only 3 investigated the ND. Results: The findings suggest that adherence to the Mediterranean and Nordic diets is generally associated with improved cognitive function and delayed cognitive decline and that adherence to both these diets can improve cognitive function. Some studies identified that higher legume consumption decreased dementia incidence, while fruits and vegetables, carbohydrates, and eggs lowered dementia prevalence. Most studies demonstrated that high MD or ND adherence was associated with better cognitive function and a lower risk of poor cognition in comparison to individuals with lower MD or ND adherence. However, some studies reported no significant benefits of the MD on cognitive outcomes, while two studies indicated that higher red meat consumption was linked to better cognitive function. Conclusion: Despite promising trends, the evidence remains varying across studies, underscoring the need for further research to establish definitive associations between diet and cognitive function. These findings highlight the essential role of dietary interventions in the prevention and management of dementia and AD, therefore offering critical insights into the underlying mechanisms by which the diet may impact brain health.

## 1. Introduction

### 1.1. Alzheimer’s Disease and AD Genetics

Alzheimer’s Disease (AD) is the predominant form of dementia, comprising 60% to 70% of reported cases. AD is a progressive disease where symptoms gradually develop over many years and, after some time, become more severe [1,2]. Initial neuronal loss in AD originates within the medial temporal lobes during the early stages of cognitive decline (CI) and subsequently progresses to the cortical areas [3]. Clinical symptoms include subtle memory lapses and difficulties with concentration and problem-solving at the early disease stages [1,2]. In the symptomatic stage, symptoms become more definite, with noticeable impairments in language, spatial orientation, and executive function, leading to challenges in daily activities and increased dependence on caregivers [4,5]. In late-stage AD, there is severe cognitive decline, with individuals losing the capacity to communicate efficiently, recognize loved ones, and perform basic daily tasks, resulting in profound functional impairment and requiring extensive support and supervision [1,2]. Dementia refers to a group of symptoms that are severe enough to significantly impact an individual’s quality of life (QoL) (https://www.alz.org). It can be categorized into several types, including AD, vascular dementia (VaD), Lewy body dementia (LBD), Parkinson’s Disease dementia (PDD), and Frontotemporal dementia (FTD). Table 1 outlines the key differences between these common forms of dementia. Numerous neuropsychological examinations, such as the mini-mental state examination (MMSE), Montreal Cognitive Assessment (MoCA), and several other tests, are used to evaluate cognitive function [6].

AD is characterized by the accumulation of amyloid-beta (Aβ) plaques and neurofibrillary tangles (NFTs) [7]. Aβ plaques are primarily composed of aggregated Aβ peptides and are hallmark neuropathological features of AD [8]. These plaques disrupt neuronal function and contribute to synaptic dysfunction and eventual neurodegeneration [7]. The build-up of hyper-phosphorylated tau leads to neurofibrillary tangle formation [9]. The deposition of NFTs is closely associated with neuronal loss in AD patients [7,9]. AD is a complex disease with both environmental and genetic predispositions involved in the disease. Familial AD (fAD) often exhibits autosomal dominant inheritance. The genes primarily involved in AD are Amyloid Precursor Protein (APP), Presenilin 1 (PSEN1), and Presenilin 2 (PSEN2), which are associated with early-onset familial AD (fAD), variations in apolipoprotein E (APOE) gene increase the risk of late-onset sporadic AD (sAD) [10]. These genetic factors contribute to the dysregulation of amyloid-beta (Aβ) metabolism, tau protein phosphorylation, and synaptic dysfunction, ultimately leading to neuronal degeneration and cognitive decline.

### 1.2. Mediterranean Diet and Its Role in AD and Dementia

The Mediterranean Diet (MD) is a prominent modifiable factor influencing the susceptibility to dementia and presents an avenue for targeted intervention in disease prevention. The promotion of healthier dietary patterns, notably the MD, has been posited as a potential strategy to attenuate dementia risk [11]. The MD is characterized by elevated consumption of vegetables, fruits, nuts, seeds, and whole grains, supplemented with a regular intake of fish and other seafood at least twice a week. Conversely, red meat and confectionery products are infrequently consumed within this dietary regimen [12]. Olive oil assumes a pivotal role as the primary cooking fat, while the intake of saturated or solid fats remains low [13].

Long-chain omega-3 polyunsaturated fatty acids, found in fatty fish such as salmon and mackerel, have been implicated in preserving cognitive function and mitigating neurodegeneration [14]. These fatty acids exert anti-inflammatory effects and support neuronal membrane integrity, potentially delaying the onset or progression of neurodegenerative diseases [14]. Additionally, moderate consumption of red wine accompanies meals. Multiple prospective cohort studies have consistently indicated a correlation between higher adherence to the MD and reduced brain atrophy, enhanced cognitive function, and a decreased risk of dementia, including AD [15]. Despite recent systematic and umbrella reviews suggesting a potential mitigating effect of strong adherence to the MD on cognitive decline [16,17], the evidence supporting its protective role against the onset of dementia remains inconclusive [18]. Additionally, the impact of a wholesome diet in potentially ameliorating an individual’s genetic predisposition toward dementia warrants attention. Previous investigations into gene–diet interactions have been limited, yielding inconsistent results, often concentrating solely on the APOE genotype [19]. However, the utilization of polygenic risk scores (PRS), consolidating information from multiple risk alleles associated with dementia and weighting them based on their respective strengths of association, has emerged as a promising avenue for predicting incident all-cause dementia [20]. This innovative approach facilitates a more comprehensive exploration of potential gene–diet interactions influencing the risk of dementia.

### 1.3. Nordic Diet and Its Role in AD and Dementia

The traditional Nordic Diet (ND) is rooted in customary Scandinavian culinary practices. It is characterized by a focus on increased consumption of vegetables, fruits, berries, fish, and whole-grain products prevalent in Nordic countries, alongside moderated intake of meat and alcohol [21,22]. A notable feature of this dietary pattern is the utilization of rapeseed oil, which contains notably higher levels of essential fatty acids, particularly linoleic acid and α-linolenic acid, compared to olive oil—a staple in the MD [23]. Berries, rich in diverse polyphenols, antioxidants, and other bioactive compounds, constitute a significant component of this diet. Primary grains such as rye, oat, and barley, commonly consumed in the form of bread and porridge, contribute substantially to dietary fiber intake.

Polyphenol-rich foods, including berries, nuts, and green tea, possess antioxidant and anti-inflammatory properties that may confer neuroprotection [24]. Polyphenols can modulate signaling pathways involved in neuronal survival and synaptic plasticity, thereby attenuating neurodegenerative processes [25,26].

While previous research has established a link between adherence to the ND and reduced cardiovascular risk factors [21,22], its potential impact on cognitive function remains largely unexplored. Given the established association between improved cardiovascular health and a decreased risk of vascular dementia [26], a hypothesis emerges suggesting that adherence to the Nordic Diet may attenuate the rate of cognitive decline in aging individuals. Furthermore, many constituents of the ND have previously exhibited associations with the preservation of cognitive abilities [27].

#### Similarities and Differences Between the Mediterranean and Nordic Diets

The MD and ND are quite similar in more ways than one, as previously mentioned, due to the high consumption of fruits and vegetables, whole grains, legumes, fatty fish, and minimally processed foods [28]. The principal difference between the two diets is the fatty acid source, where EVOO is the main dietary fatty acid in the MD, while for the ND, this is rapeseed oil [28]. MD adherence and its role in preventing chronic non-communicable diseases has been extensively studied and validated for its cognitive benefits, via longitudinal epidemiological studies and randomized controlled trials to explore its unique impact and beneficial effects [28]. Furthermore, the ND provides a culturally relevant alternative for populations in Northern Europe, where traditional MD components such as EVOO and certain fruits and vegetables may not be readily available. This, however, underscores the importance of dietary personalization in promoting adherence and achieving neuroprotective effects. The health weapon for both diets is the high consumption of fruits, vegetables, nuts, and legumes. However, the type of fruit or vegetable for each diet varies, as the MD features warm-weather fruits and vegetables such as greens, tomatoes, eggplants, figs, dates, and pomegranates, whereas the ND features starchier fruits and vegetables such as carrots, turnips, beets, apples, plums, and pears [28]. In addition, the MD includes grains such as whole wheat bread and pasta, whereas the ND includes barley, oats, and rye [28]. Nevertheless, there are limited studies investigating the potential benefits of ND adherence, including the role of phytochemicals and essential fatty acids in slowing cognitive decline. Nevertheless, the lack of robust, longitudinal studies limits the ability to draw firm conclusions in the context of AD and dementia [28,29,30,31]. In addition, both diets interplay with genetic factors, particularly the APOE genotype, which remains an underexplored avenue. Incorporating PRS in future research could enhance our understanding of gene–diet interactions [1,7]. Lastly, methodological standardization in dietary assessments and cognitive measurements could improve cross-study comparability.

Despite the wealth of studies demonstrating the protective role of the MD in neurodegenerative diseases such as AD, the effects of the ND on AD and dementia have not been extensively investigated. The state of the art of this systematic review relies on the comprehensive investigation of the MD and ND and their beneficial effects on dementia and AD. Moreover, based on our research, there is a lack of studies that include an overview of studies on the health benefits of the MD and ND in dementia and AD.

Nevertheless, this investigation needs to be preceded by a review of all the relevant studies published to date to streamline any existing results, therefore highlighting research significance. The aim of this review is to summarize studies evaluating the effects of MD and ND adherence, dietary intake, and extra virgin olive oil (EVOO) in AD and dementia populations.

## 2. Materials and Methods

### 2.1. Search Strategy and Study Selection

**Figure 1:**
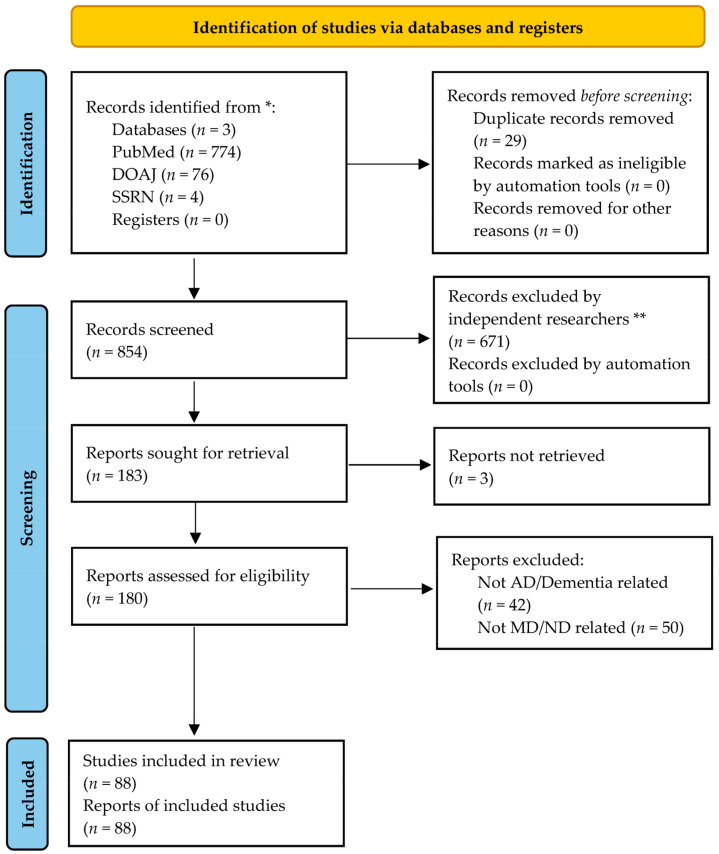
PRISMA 2020 flow diagram for new systematic reviews indicating the identification, screening, eligibility, and studies included, as well as the number of excluded studies from the review. * If feasible, report the number of records identified from each database or register searched. ** If automation tools were used, indicate how many records were excluded by a human and how many were excluded by automation tools.

To examine the potential correlation between AD, dementia, and dietary variables, specifically regarding MD adherence and the ND in human subjects, a literature review was conducted utilizing electronic databases such as PUBMED, Directory of Open Access Journals (DOAJ), and SSRN. The search spanned studies published from 2006 to 2024. The following search terms were employed: “Dementia AND Mediterranean Diet”, “Dementia AND Nordic Diet”, “Alzheimer’s Disease AND Mediterranean Diet”, and “Alzheimer’s Disease AND Nordic Diet”. Studies that were related to the other forms of dementia, such as VaD, LBD, PDD, and FTD, were excluded from this review, as we were only interested in the AD dementia subtype. In addition, studies that included both sporadic AD and familial AD were included in this systematic review. A total of 854 articles were retrieved from the search, 29 of which were duplicates. The abstracts were screened independently by two investigators; if the studies were relevant, full articles were then reviewed. Data were then extracted from the identified studies. The cited references of the included studies were further searched for any additional relevant publications. The article selection process is outlined in (Figure 1). The PRISMA checklist is in the Supplementary Materials (Table S1). The following systematic review follows the PRISMA guidelines, and no registration information is applicable. The Population, Intervention, Comparison, and Outcome (PICO) of this review is defined as the AD and dementia or healthy elderly population (P) to observe what is the effect of the MD and ND (I) in improving cognitive function and delaying dementia or AD onset (O) in comparison to individuals with lower adherence to the MD and ND (C).

### 2.2. Assessment of Risk of Bias in Included Studies

Quality assessment tools are used to assist researchers and reviewers in focusing on concepts vital for the study’s validity. This is performed in order to avoid risk bias and obtain a deeper understanding of the limitations of the selected studies. The quality of eligible studies was critically assessed using the NIH study quality assessment tools https://www.nhlbi.nih.gov/health-topics/study-quality-assessment-tools (accessed on 8 November 2024) for (i) case–control studies, (ii) observational cohort, (iii) longitudinal, and (iv) cross-sectional studies. The Cochrane quality assessment of risk bias https://methods.cochrane.org/risk-bias-2 (accessed on 4 December 2024) was used for randomized controlled trials (RCTs).

## 3. Results

### 3.1. Search Results

The ensuing study types were included in the review: (i) cross-sectional studies, (ii) RCTs, (iii) longitudinal, prospective, and retrospective studies, (iv) cohort studies, (v) case–control studies, and (vi) population-based studies. In the process of refining the selection of studies, several stringent exclusion criteria were rigorously applied to ensure study relevance, quality, and applicability of the data. These include studies (i) involving non-human participants, specifically animal models, (ii) non-English publications, (iii) studies that did not explicitly investigate the relationship between the MD or ND and AD or dementia were classified as irrelevant for the purpose of this review, (iv) systematic reviews, meta-analyses, narrative reviews and conference proceedings, and (v) studies were other forms of dementia such as VaD, LBD, PDD, and FTD. The exclusion of these articles was intended to prevent duplication of findings and to ensure that the review focused solely on original research studies.

### 3.2. Study Characteristics of Included Studies

An overview of the studies included in the review is illustrated in Supplementary Materials (Table S2). This includes (i) types, (ii) study references, (iii) study country, (iv) study participants, (v) age range, and (vi) study type. A total of 88 unique and relevant studies were included in the review. Eighty-five (Table 2) original articles were included for the association of MD adherence in AD and dementia. Three studies (Table 3) were included for the association of ND adherence in AD and dementia. As outlined below, most studies support the hypothesis that specific dietary intake can delay disease progression and improve cognitive function. Alternatively, some studies [32,33] indicated that the MD has no beneficial effect on AD, dementia, or MCI participants in terms of their cognitive function. While one study [34] identified that daily consumption of cheese and red wine and weekly consumption of lamb may improve long-term cognition, the following study contradicts previous studies that dairy, red wine, and meat consumption should be of moderate consumption, a summary of all the study results can be seen in Supplementary Data S1. Lastly, a study by [35] found inconsistent findings that MD adherence improved cognitive function and decreased cognitive decline.

### 3.3. Quality Assessment of Risk Bias of Studies

To assess the quality of the studies included, a quality assessment of risk bias was performed, and studies were categorized based on the study type (case–control, cohort studies, and RCTs). Quality assessment of risk bias using Cochranes was performed for RCT studies, Supplementary Materials (Table S3). The NIH quality assessment of risk bias was used for the case–control (Table S4), observational cohort, longitudinal, and cross-sectional studies (Table S5).

## 4. Discussion

Over the past decade, there have been various studies illustrating the importance of maintaining a healthy lifestyle through physical activity, cognitive-related activities, and healthy eating that includes MD or ND adherence. These factors have been shown to delay cognitive decline, MCI, and dementia or provide protection against the occurrence of chronic diseases such as AD. Numerous studies have evaluated the associations between the Mediterranean [6,26,27,36,37,38,39,40,41,44,46,47,48,49,50,54,56,57,59,60,61,62,63,65,66,67,68,69,70,72,73,74,75,76,78,79,80,81,82,83,84,85,86,87,88,89,90,91,92,93,94,95,97,98,99,100,102,103,104,105,107,108,109,110,111,112,113,114,115], Nordic [29,31,116,117], and Western diets [52,79,118,119,120] and diseases. There is a consensus on how dietary intake and patterns can explain the etiology of common diseases such as cardiovascular, metabolic, and neurodegenerative diseases. Moreover, there is growing evidence indicating the benefits of the MD and ND in AD and dementia.

The findings presented in this systematic review highlight the nuanced and complex relationships between dietary patterns, specifically that of the MD and ND diets, on cognitive function, dementia onset, progression, and management. The extensive body of evidence accumulated from the 88 studies strongly supports the neuroprotective benefits of these dietary patterns, suggesting their potential to reduce the risk of cognitive decline and delay the onset of dementia and AD. These findings emphasize the pivotal role of dietary interventions in the prevention and management of dementia and AD, therefore providing critical insights into the underlying mechanisms by which the diet may impact brain health.

### 4.1. Mediterranean Diet, Alzheimer’s Disease, and Cognitive Health

The findings corroborate the probable neuroprotective role of the MD in AD and dementia-related diseases. Key aspects of the MD include the high intake of fruits, vegetables, nuts, seeds, whole grains, and olive oil consumption, in combination with moderate fish and red wine consumption, which contribute to the beneficial outcomes of MD adherence [121,122]. The MD’s effectiveness is accredited to quite a few mechanisms, such as anti-inflammatory effects, were components like omega-3 fatty acids obtained from fatty fish and polyphenols from olive oil exhibit anti-inflammatory properties that may reduce neuronal cell death [122,123]. The MD is rich in antioxidants such as vitamin E, known to combat reactive oxidative species (ROS), which contribute to DNA damage, distribution of the lipid bilayer, and neuronal cell death and degeneration [122]. Moreover, ROS are known contributors to AD pathology. Lastly, improved cardiovascular health associated with the MD indirectly supports brain health as vascular contributions such as uncontrolled blood pressure and cholesterol are known risk factors for dementia [122,123,124]. Countless studies have reported significant associations between higher MD adherence and improved cognitive outcomes, delayed cognitive decline, and a decreased risk of developing AD and dementia, as reported in this study due to the high consumption of fruits, vegetables, legumes, and EVOO. Furthermore, a combination of high MD adherence, along with physical activity [45], was associated with better cognitive performance. This suggests that a healthy lifestyle could reduce cognitive decline [45]. Notable findings include cognitive improvements and improved executive function and global cognition in individuals when MD adherence is supplemented with EVOO and nuts. Moreover, in long-term interventions involving a combination of MD + EVOO, study participants showed improved cognitive test scores in both global and specific domains such as memory. In addition, longitudinal cohort studies highlighted between 24 and 48% decrease in AD risk among participants in the higher MD adherence tertiles. It is noteworthy to mention that these effects were independent of vascular comorbidities, thus suggesting direct neuroprotective roles of the MD.

In addition, a study by Zhang et al. [125] investigated meat consumption and the risk of dementia incidence in 493,888 U.K. biobank participants [125]. Over the years, meat consumption has been correlated with dementia risk; however, the serving amount and meat type related to dementia risk have been poorly understood [125]. Overall, the study identified that an additional 25 g/day intake of processed meat consumption was associated with an increased risk of dementia and AD, while a 50 g/day increment in unprocessed red meat consumption was correlated with a decreased risk of dementia and AD [125]. It is noteworthy to mention that although processed meat consumption was associated with an increased risk, the APOE ε4 allele increased dementia risk between three and six times without modifying dietary associations significantly [125].

High sugar intake is another dietary component associated with an increased risk of dementia and AD development [113]. A study by Agarwal [79] investigated whether high sugar intake is correlated with an increased risk of dementia in older adults [79]. It was observed that 118 participants developed dementia during follow-up [79]. In comparison to participants with the lowest total sugar intake, those with the highest intake developed AD, on average, 7.1 years earlier. Therefore, a higher percentage of calories from sugar intake was correlated with an increased risk of AD [79].

### 4.2. Nordic Diet, Alzheimer’s Disease, and Cognitive Health

The ND shares similarities with the MD, where it emphasizes whole, minimally processed foods native to the Nordic countries of Sweden, Denmark, Norway, Finland, Iceland, and Greenland. The ND includes the consumption of berries, vegetables, whole grains, and fatty fish legumes [28]. Berries, vegetables, and legumes are rich in micro- and macronutrients, antioxidants, and phytochemicals. Whole grains offer a low glycemic index that assists in reducing oxidative stress and inflammation, while fatty fish (salmon, bluefin tuna, anchovies, herring sardines, and mackerel) are high in omega-3 fatty acids [28]. The main MUFA consumed is rapeseed oil. It is an excellent source of vitamin E and consists of alpha-linolenic acid (ALA), which has been shown to have health benefits correlated with lowering blood pressure and reducing the likelihood of a heart attack. However, the role of ALA in cognition is still in the early research stages, but it displays promising evidence for hindering cognitive impairment and promoting cognitive health [126,127]. Erucic acid is the main phenolic compound in rapeseed oil; it is an omega-9 MUFA [128]. The ND’s reliance on rapeseed oil, as opposed to EVOO, raises questions regarding differences in the neuroprotective effects of specific lipid profiles [28,29,30]. Moreover, while berries and whole grains offer unique phytochemicals, their specific contribution to brain health in the ND context warrants further investigation.

Despite the potential antioxidant, anti-inflammatory, neuroprotective, and cardiovascular benefits, the evidence linking the ND to cognitive outcomes remains sparse, with only three studies in the review examining this relationship. The potential of the ND in terms of cognitive function has been demonstrated through studies that have observed that high ND adherence is associated with at least 4 years longer life without dementia [31]. Moreover, an active lifestyle that involves physical activity and a healthy dietary intake has been shown to significantly enhance the protective effect of these factors on cognitive function and reduce the risk of cognitive decline [30]. These studies provided some indications of the cognitive benefits of the ND in AD and dementia; however, the limited sample size and diversity hinder definitive conclusions.

### 4.3. Micronutrients and Macronutrients and Their Role in Alzheimer’s Disease and Dementia

Nutrients, by definition, are vital molecules necessary for the proper functioning of the human body. Most of these cannot be produced within the body and must be acquired through dietary intake [114]. The brain, being an organ with elevated metabolic activity and rapid nutrient turnover, is particularly reliant on a consistent supply of essential nutrients [114]. The relationship between vitamins and cognitive function is a topic of considerable interest in both clinical and research domains due to the profound implications of vitamin deficiencies on cognitive health [129]. A plethora of vitamins, encompassing the B complex group, vitamin D, and vitamin E, have emerged as pivotal players in modulating cognitive processes and ameliorating cognitive decline (Morris et al., 2007). Notably, vitamin B12, essential for methylation reactions and myelin sheath formation, has been intricately linked to cognitive impairment when its levels are inadequate [129]. Similarly, vitamin D, renowned for its regulatory roles in calcium homeostasis and neuronal growth, has garnered attention for its potential impact on cognitive function. Observational studies have suggested an inverse relationship between vitamin D status and cognitive decline, indicating that this vitamin may play a protective role against cognitive impairment [101]. Moreover, vitamin E, recognized for its potent antioxidant properties, has demonstrated promise in shielding against age-related cognitive decline and neurodegenerative disorders [130]. Through its ability to scavenge free radicals and mitigate oxidative stress in the brain, vitamin E emerges as a potential therapeutic agent for preserving cognitive function with advancing age [130]. A comprehensive understanding of the intricate interplay between vitamins and cognitive function holds substantial implications for the formulation of interventions aimed at sustaining cognitive health throughout the lifespan.

Calcium serves as a critical signaling molecule in neuronal communication and synaptic plasticity, fundamental processes underlying learning and memory [131]. Calcium ions regulate neurotransmitter release, intracellular signaling cascades, and gene expression within the brain, thereby influencing cognitive processes such as memory formation and information processing [132,133]. Maintaining calcium homeostasis is paramount for preserving neuronal viability and mitigating the risk of neurodegenerative diseases associated with cognitive decline [132,133]. Dysregulation of calcium levels has been implicated in the pathogenesis of AD and other neurodegenerative diseases [24]. Studies have underscored the critical role of calcium signaling in neuronal function and survival, emphasizing its significance in the context of neuroprotection and disease prevention [24]. Deficiencies in calcium intake or dysregulation of calcium signaling have been implicated in cognitive impairment and neurodegenerative disorders, highlighting the importance of adequate calcium levels for cognitive health [24].

### 4.4. Implications for Public Health and Interventions

Encouraging adherence to these dietary patterns may assist as a feasible and non-invasive strategy for delaying and preventing cognitive decline. Tailored interventions, such as community-based programs and dietary counseling, may promote the implementation of these diets in populations at risk of AD, with the hope of this applying to other NDs. However, personalization based on genetic predispositions, dietary accessibility, and cultural dietary preferences is essential and could enhance adoption and effectiveness globally. Promoting MD adherence and investigating the ND’s efficacy could lead to personalized nutritional strategies to mitigate dementia risk. The following points to the need for further longitudinal and randomized controlled studies to refine our understanding of the neuroprotective benefits of these diets. Moreover, integrating dietary-based research with genetic, -omics, biochemical, and lifestyle factors will further provide a holistic approach to combating AD and dementia.

### 4.5. Strengths, Limitations, and Future Directions

This systematic review is strengthened by the inclusion of (i) a diverse range of study designs, from RCTs to longitudinal cohort and cross-sectional studies, which collectively provide a robust investigation of the effects of the MD and ND on cognitive health across various populations of cognitively healthy older individuals, elderly men, and post-menopausal women at risk of dementia, AD, SCD, MCI, and CI study participants; (ii) the use of a wide array of neuropsychological assessment tools, such as the MMSE, RAVLT, MoCA, AF, CASI, and several cognitive tools. Additionally, advanced neuro-imaging techniques such as MRI and PET, to further enhance the validity and reliability of the findings to monitor brain changes; (iii) focus on nutrient-rich foods such as EVOO, nuts, and fatty fish, this underscores modifiable dietary factors that may be integrated into preventive strategies; and (iv) the consistent observation of improved cognitive outcomes with higher MD adherence across multiple studies bolsters the argument for its neuroprotective effects. An additional strength of the study lies in (iii) the large sample sizes included for each study, allowing for the identification of significant statistical results and associations between the MD and ND, as well as preventive outcomes of cognitive decline in individuals. Lastly, numerous studies included (iv) follow-up of study participants, allowing for more confidence in inferring whether diet affects the clinical phenotype.

However, several limitations must be acknowledged within our study. A significant limitation is the (i) reliance on self-reported dietary data, and recall bias may not reflect participants’ dietary intake. This limitation can affect the precision of the associations between diet and cognitive outcomes. Additionally, the variability in dietary assessment methods, such as FFQs and dietary recalls, across different studies poses challenges in comparing and synthesizing results. The (ii) heterogeneity in study populations, including differences in age, sex, genetic background, and baseline cognitive function, presents another limitation. These variations may impact the observed effects of diet on cognitive health and limit the generalizability of the findings. Although many studies adjust for potential confounders such as age, education, and physical activity, residual confounding factors, such as socioeconomic status, which can impact both dietary choices and access to healthcare, are not always adequately addressed, (iii) potential interaction between diet and genetic factors, particularly the APOE genotype, is an area that requires further exploration, and (iv) the exclusion of other dementia subtypes such as VaD, LBD, PDD, and FTD as the focus of the review was the dementia subtype of AD. The inconsistency in findings regarding gene–diet interactions highlight the need for more research to clarify how genetic predispositions impact the effectiveness of dietary interventions in preventing cognitive decline.

Future directions include (i) cross-cultural studies to validate findings across diverse populations, (ii) integration of genetic and dietary data for personalized nutritional approaches, and (iii) comprehensive assessments of the ND to evaluate its beneficial effects and role in delaying disease onset and disease management.

## 5. Conclusions

This systematic review consolidates robust evidence, supporting the significant role of dietary patterns, specifically the MD and ND, in mitigating the risk of cognitive decline and delaying dementia and AD onset. Study findings consistently highlight the neuroprotective effects of the MD, which is rich in antioxidants, MUFAs, and phenolic compounds, which are recognized for their anti-inflammatory, antioxidant, and neuroprotective properties, which collectively contribute to the reduction in pathological processes such as oxidative stress, inflammation, and Aβ accumulation, central to AD pathogenesis. While the MD demonstrates robust associations with cognitive benefits, the ND embodies a promising yet unexplored avenue.

Despite variability in findings across different studies and populations, the overall evidence underscores the potential of these dietary interventions in promoting cognitive health and reducing the risk of neurodegenerative diseases, including AD and dementia.

The observed interactions between diet, genetic predispositions, and lifestyle factors further emphasize the need for personalized dietary recommendations to optimize cognitive outcomes. As the global population ages and the burden of AD and dementia continues to rise, the identification and implementation of effective, accessible, and sustainable preventive strategies are of paramount importance. The integration of the MD and ND into public health recommendations could play a critical role in combating cognitive decline, improving the quality of life for aging populations, and reducing the global burden of neurodegenerative diseases. Further research and interdisciplinary collaborations are essential to advancing our understanding of these dietary patterns and their role in the prevention and management of cognitive decline and neurodegenerative diseases.

## Figures and Tables

**Table 1:** The differentiation between common forms of dementia.

	AD	VaD	LBD	PDD	FTD
Course	Subtle onset and gradual progression	Based on location of microscopic bleeding and extent of cerebrovascular event (CVE)	Subtle onset and gradual progression	Subtle onset and gradual progression	Subtle onset and gradual progression
Presentation	Early disease stages: Memory loss and impaired learning Moderate to severe stages: Language and visuospatial deficits	Temporal relationship between CVE and cognitive impairment onset Subcortical ischemic vascular disease: Dysexecutive function	Fluctuating cognitive and functional impairment with Parkinsonism, sleep disorder, and visual hallucinations Cognitive symptoms start before or concurrently with motor symptoms Resting tremor less frequent	1 year after motor symptoms, cognitive decline starts Movement impairment: Resting tremor, rigidity, bradykinesia Cognitive impairment: Bradyphrenia, inattention, executive, visuospatial dysfunction	bvFTD: Behavioral disinhibition, apathy, loss of sympathy/empathy, perseverative stereotyped speech, dietary changes, OCD behavior PPA: Loss of word memory May present with both types

bvFTD: behavioral variant, OCD: obsessive compulsive disorder, PPA: language variant.

**Table 2:** Characteristics of studies investigating the association of the Mediterranean Diet, dietary patterns, and intake in Alzheimer’s Disease, dementia, and at-risk individuals.

First Author, Year (Country)	Study Type, Subjects (*n*), and Ethnicity	Mean Age at Sample Collection (Years)	Sample Type	Cognitive Function Assessment	Exposure (Dietary Consumption, Dietary Patterns)	Clinical Outcome, Analysis, and Effect Estimation	*p*-Value	Cofounders	Clinical Conclusions
Chan et al., 2013 (China) [36]	Cross-sectional **Chinese men** (*n* = 1926) **Chinese women** (*n* = 1744) **Total** (*n* = 3670) **CI:** **Men** (*n* = 221) **Women** (*n* = 656) **Ethnicity** Chinese	>65	NR	Cognitive function assessed by Community Screening Instrument for Dementia (CSI-D)	Dietary data collected using a validated FFQ. **A priori dietary pattern:** MD score (MDS) A posteriori dietary patterns: “Vegetables–fruits” pattern “Snacks–drinks–milk products” pattern “Meat-fish” pattern	**Women:** **Vegetables–fruits pattern:** Adjusted OR = 0.73 (95% CI: 0.54–1.00) **Snacks–drinks–milk products pattern:** Adjusted OR = 0.65 (95% CI: 0.47–0.90). **Men:** No dietary patterns significantly associated with cognitive impairment risk.	0.018 * 0.003 *	NR	Higher vegetables–fruits, snacks–drinks–milk products pattern scores associated with reduced risk of cognitive impairment in older Chinese women.
Charisis et al., 2021 (Greece) [37]	Longitudinal study Non-demented individuals (*n* = 1046) **Follow-up** **Dementia cases** (*n* = 62) **Ethnicity** Greek	73.1 ± 5.0	NR	Comprehensive neuropsychological assessment (memory, language, attention-speed, executive functioning, visuospatial perception)	MD score from FFQ	72% lower risk of dementia in higher MD adherence quartile compared to lower. 10-unit increases in MD score offsets 1 year of cognitive aging.	0.013 *	Age Sex Education BMI Energy intake ApoE genotype Comorbidities Physical activity	Higher MD adherence associated with reduced risk of dementia and cognitive decline.
Valls-Pedret et al., 2015 (Spain) [38]	Parallel group RCT Cognitively healthy volunteers **Female** (*n* = 233) (52.1%) **Follow-up** (*n* = 334) **Total** (*n* = 447) **Follow-up** 6 y **Ethnicity** Spanish	66.9	Blood Urine	MMSE RAVLT Animal Fluency Test Digit Span VPA Color Trail Test	MD supplemented with EVOO oil (1 L/week) or mixed nuts (30 g/day)	MD+EVOO group scored better on RAVLT and Color Trail Test part 2 vs. controls. MD + EVOO vs. controls. Frontal cognition improved significantly in the MD plus olive oil group.	0.049 * 0.04 * 0.04 * 0.003 * 0.005 *	Age Sex Education APOE ε4 genotype Smoking BMI Energy intake Physical activity	MD supplemented with EVOO or nuts associated with improved cognitive function. Global cognition improved significantly in the MD plus olive oil group.
Kuhn et al., 2021 (South Africa) [39]	RCT Cognitively intact elderly participant **Intervention group** (*n* = 31) **Control group** (*n* = 26) **Total** (*n* = 57) **Ethnicity** South African	72 ± 7	NR	CASI	Fish intake (2.2 g omega-3 PUFA daily) a modified MIND diet. **Intervention group** Canned pilchards and fish spread **Control group** Canned meatballs and texturized soy every 12 weeks	Intervention group had significantly higher post-intervention CASI score than controls. Higher intake of omega-3 PUFA, RBC EPA, and DPA content in intervention group.	0.036 * 0.004 * 0.013 *	Baseline scores Education Omega-3 PUFA supplementation	12 weeks of additional fish intake improved cognition in resource-limited elderly people.
Zupo et al., 2022 (Italy) [40]	Cross-sectional retrospective study **Normal cognition group** (*n* = 467) **MCI** **group** (*n* = 117) **Total** (*n* = 584) **MICOL3 (M3):** GreatAGE study **(MICOL4, M4):** Participants from MICOL3 over 64 years **Ethnicity** Italian	**M3** 66.5 ± 6.1 **M4** 73.9 ± 6	NR	MMSE	Plant-based diet, including coffee, vegetables, and vitamin A sources	Plant-based foods inversely associated with cognitive impairment, alcohol consumption detrimental, and red meat to be beneficial.	0.001 * 0.03 * 0.04 *	Age Sex Smoking Education BMI APOE ε4 Physical activity	Traditional MD pattern, low alcohol consumption helps prevent/delay cognitive impairment.
Martínez-Lapiscina et al., 2013 (Spain) [41]	RCT **PREDIMED Navarra Center** **Total** (*n* = 1055) **MD + EVOO diet** (*n* = 351) **MD + nuts diet** (*n* = 352) **MD + low-fat control diet** (*n* = 352) **After follow-up** **MD + EVOO** (*n* = 91) **MD + Nuts** (*n* = 88) **MD + Low Fat** (*n* = 89) **Total** (*n* = 268) **Follow-up** 6.5 y **Ethnicity** Spanish	74.1 ± 5.7	Blood	MMSE RAVLT VPA ROCF CDT TMT	Dietary habits evaluated using a validated 137-item MD supplemented with EVOO or mixed nuts vs. low fat control diet	MD + EVOO group significantly better performance compared to controls in visual and verbal Memory, ROCF delayed, fluency (FAS and Digit Forward Test). **Odds ratio for MCI:** MD + EVOO group had significantly lower odds of MCI compared to control.	0.008 * 0.033 * 0.047 * 0.012 * 0.005 * 0.024 *	Sex Age Education APOE BMI Physical activity Smoking Family history of MCI/dementia Hypertension Alcohol consumption Dyslipidemia Diabetes Total energy intake	Long-term intervention with an EVOO-rich MD resulted in better cognitive function and lower MCI compared with a control diet.
Haring et al., 2016 (USA) [32]	Prospective cohort study Post-menopausal women **Total** (*n* = 6425) **Follow-up** 9 y **MCI:** (*n* = 499) **PD:** (*n* = 390) **Ethnicity** NR	65–79	Blood	MMSE Consortium to Establish a Registry for Alzheimer’s Disease (CERAD) battery, others	**Dietary patterns assessed by FFQs**: aMED, HEI-2010, AHEI-2010, DASH	No statistically significant relationships across quintiles of aMED, HEI-2020, DASH, and AHEI-2020 scores and MCI or PD.	0.30 0.44 0.23 0.45	NR	Dietary patterns of aMED, HEI-2010, AHEI-2010, or DASH not associated with cognitive decline. Adherence to a healthy dietary pattern did not modify risk for cognitive decline.
Muñoz-García et al., 2022 (Spain) [42]	Prospective cohort Subgroup of Segui-miento Universidad de Navarra (SUN) cohort of university graduates >55 years old **Total** (*n* = 803) **Follow-up** 2 and 6 yrs **Ethnicity** Spanish	61 ± 6 years	Saliva	Spanish Telephone Interview for Cognitive Status (STICS-m)	Dietary patterns assessed by a validated 136-item FFQ WDP and MDP	WDP associated with negative STICS-m changes (−0.80 points, 95% CI: 1.51 –0.08). MDP associated with positive STICS-m changes (+0.71 points, 95% CI: 0.15–1.26).	0.03 * 0.01 *	NR	MDP associated with less cognitive decline, lowering dementia incidence. WDP associated with a greater cognitive decline increasing dementia incidence.
Tzekaki et al., 2019 (Greece) [43]	RCT **AD** (*n* = 30) **Healthy** (*n* = 16) **MCI with EVOO therapy** (*n* = 36) **MCI without therapy** (*n* = 37) **Total** (*n* = 108) **Ethnicity** Greek	NR	Serum levels of fibrinolytic factors, tau, Aβ amyloid fragments, and MDA	NR	EVOO given for 1 year	Reduced levels of PAI-1 and a2-antiplasmin in MCI patients with EVOO therapy. Aβ1-42/Aβ1-40 ratio like healthy individuals, decreased tau protein levels and MDA levels.	NR	NR	EVOO therapy may prevent progression of MCI to AD by decreasing fibrinolytic factors, hallmarks of AD and MDA.
Walters et al., 2018 (USA) [44]	Prospective longitudinal study Cognitively healthy, middle-aged participants (30–60 yrs) (*n* = 70) **Ethnicity** Caucasian	49 ± 8	MRI PET (FDG and PiB)	Global cognitive z-score from multiple tests	MD adherence Intellectual Physical activity Vascular risk measures	Lower MD adherence associated with faster FDG decline in PCC and in frontal cortex. Higher baseline homocysteine linked to faster cognitive decline.	0.048 *	Age Sex APOE status BMI Physical activity	MD adherence impacts glucose metabolism in the brain. Higher homocysteine predicts cognitive decline.
Mosconi et al., 2018 (USA) [3]	Cross-sectional, observational Middle-aged adults, broader New York City area **Total** (*n* = 116) **Follow-up** 3 y **Ethnicity** NR	50 ± 8 **High MD adherence** 49 (9) **Low MD adherence** 50 (9)	MRI	MMSE Memory Executive function Language tests	MD High and low MD adherence	MD and insulin sensitivity was positively associated with MRI-based cortical thickness (βs ≥ 0.26) **Insulin sensitivity:** (βs ≥ 0.58) **EC thickness:** Variance in memory **Intellectual enrichment:** Associated with better cognition (βs ≥ 0.25 **Overweight:** Associated with lower cognition (βs ≥ −0.22).	≤0.008 * ≤0.008 * ≤0.001 * ≤0.001 * ≤0.01 *	Age Sex APOE status	MD and insulin sensitivity positively impact brain structure, intellectual enrichment, and obesity impacts cognitive performance.
Blumenthal et al., 2017 (USA) [45]	Cross-sectional study. Older adults with CIND **Total** (*n* = 160) **Ethnicity** Caucasian (52%) African American (46%) Other (1%)	65.4 ± 6.8,	Blood (hsCRP)	**Verbal memory:** HVLT-R, Animal Naming Test **Visual memory:** CFT **Executive Function/processing speed:** Stroop Test Digit Span COWA TMT DSST Ruff 2 and 7 Test	FFQ and 4-day food diary to assess DASH diet and MD adherence	Physical activity/ aerobic fitness associated with better executive function, processing speed, and verbal memory. DASH diet associated with better verbal memory. MD adherence not related to any cognitive domain, executive function/processing speed, verbal, and visual memory. Verbal memory and DASH diet were influenced by low total dietary fat intake.	<0.05 <0.05 0.018 * 0.901 0.167 0.978 0.053	Age Education Sex Ethnicity Family history of dementia Total caloric intake Chronic use of anti-inflammatory medication	Higher physical activity, aerobic fitness, and DASH diet adherence associated with better cognitive performance. Suggesting healthy lifestyles could reduce cognitive decline.
Chen et al., 2021 (USA) [46]	Cohort study Women, free of dementia at baseline (65–79 yrs old) **Total** (*n* = 1302) **Follow-up** 3 y **Ethnicity** Non-Hispanic White	70 years ± 3.6	MRI	3MS	FFQ, MIND diet adherence, and particulate matter with aerodynamic diameter (PM2.5) exposure	WMV positively associated with higher MIND scores, PM2.5 exposure negatively associated with WMV, interaction observed. Higher adherence to a MIND-like diet associated with greater WMV, protective effect against PM2.5 exposure.	<0.001 * <0.001 *	Intracranial volume Age Race/ethnicity Education Smoking Alcohol consumption BMI Physical activity WHI-HRT treatment Total energy intake	Higher adherence to a MIND-like diet associated with greater WMV, protective effect against PM2.5 exposure. PM2.5 exposure associated with lower MRI-based WMV, an indication of brain aging, only among women whose usual diet was less consistent with the MIND-like dietary pattern at baseline.
Olsson et al., 2015 (Sweden) [47]	Cohort study Elderly men **Total** (*n* = 1138) **Ethnicity** Swedish	**At baseline** (age 71): (*n* = 1104) participants **Survived to age 85:** (*n* = 625) participants **Re-examined at age 87:** (*n* = 369)	Blood	NR	Dietary patterns 7-day records	(Modified MD score) mMDS was not associated with dementia diagnosis.	NR	Age Education Physical activity Smoking Alcohol consumption BMI Cardiovascular disease Energy intake	No strong associations between dietary patterns and cognitive dysfunction. MD may have a potentially beneficial effect on a subpopulation.
Franzon et al., 2017 (Sweden) [48]	Cohort study Healthy Swedish men (*n* = 1104) **Follow-up** 16 years (*n* = 369) **Ethnicity** Swedish	**Baseline:** 71 (69.4–74.1) **Follow-up:** 87 (84.8–88.9)	NR	MMSE	MD adherence, lifestyle factors, cardiovascular risk factors	Independent aging was associated with never smoking (vs. current) (OR) = 2.20, 95% CI = 1.05–4.60) and high (vs. low) adherence to an MD (OR = 2.69, 95% CI = 1.14–6.80).	NR	Age Physical activity Education Cardiovascular risk factors	Never smoking, high MD adherence, maintaining a healthy weight were associated with survival and independent aging at age 85 and older.
Zhao et al., 2020 (USA) [49]	Longitudinal Cohort Study Washington Heights–Inwood Columbia Aging Project (WHICAP) cohort **Healthy older adults** **Female** (*n* = 1204) **Male** (*n* = 555) **Total** (*n* = 1759) **Follow-up** **AD cases** (*n* = 329) **Ethnicity** Multiethnic	≥65 years	Blood	DSM-IV criteria for dementia diagnosis	61-item FFQ to assess vitamin D intake from food sources	**Cox hazard regression:** Highest tertile of vitamin D intake from food sources had decreased AD/dementia risk compared with lower tertile (HR: 0.72, 95% CI: 0.54–0.97).	0.030 *	Age Sex Race/ethnicity Education APOE-ε4 Physical activity Hypertension Diabetes Cardiovascular disease Smoking	Higher vitamin D intake associated with decreased risk of dementia.
Corley et al., 2020 (UK) [50]	Prospective cohort Dementia-free subjects **Total** (*n* = 863) **Follow-up** 73, 76, 79, and 82 years of age **Ethnicity** NR	70 years	NR	Multiple cognitive tests across 4 domains (visuospatial ability, processing speed, memory, verbal ability) and global cognitive function	FFQ MD pattern and traditional pattern	Higher MD adherence at baseline associated with better verbal ability. Higher adherence to traditional diet associated with lower verbal ability. MD associated with steeper decline in verbal ability over 12 years.	0.009 * <0.001 * 0.008 *	Age Sex Childhood IQ ApoE ε4 status Smoking Physical activity Marital status Socioeconomic status	Higher MD adherence at baseline associated with better verbal ability at age 70, but not with reduced risk of cognitive decline over 12 years.
Martín et al., 2018 (Spain) [51]	Comparative study **AD patients** (*n* = 75) **Healthy controls** (*n* = 267) **Ethnicity** NR	**AD:** 77.5 ± 7.7 **Controls:** 73 ± 7.1	NR	NR	MD adherence (PREDIMED score) Gustatory function tests	Patients had lower BMI and weight, higher sleep hours, lower MD adherence impaired gustatory function in detecting salty flavor and recognizing different taste.	0.02 * 0.001 * 0.001 * 0.004 * 0.014 * NS	Age Sex Physical activity	AD patients had worse outcomes regarding BMI, weight, gustatory function, and lifestyle habits compared to controls.
Samuelsson et al., 2021 (Sweden) [52]	Population-based cross-sectional study **H70 Birth Cohort Study** Dementia-free older adults **Total** (*n* = 269) **Ethnicity** NR	70	CSF for Aβ42 Aβ40 total tau (t-tau) *p*-tau	MMSE	WD pattern, MD/prudent dietary pattern, high protein and alcohol pattern, high total and saturated fat pattern	Higher adherence to WD pattern associated with increased odds of having t-tau (OR: 1.43; 95% CI: 1.02 to 2.01) and preclinical AD (OR 1.79; 95% CI 1.03 to 3.10).	0.04 * 0.04 *	Sex Energy intake BMI Educational Physical activity	Higher WD adherence associated with pathological levels of t-tau. No associations found for other dietary patterns or biomarkers.
Chen et al., 2021 (Australia) [53]	Longitudinal study Community-dwelling non-demented individuals (*n* = 1037) **Female** (55.2%) (*n* = 572) **Follow-up** 6 y **Ethnicity** NR	**Baseline:** 70–90	NR	MMSE	DGES v2-FFQs MD, DASH diet, prudent healthy diet, Western diet	MD and DASH diet positively linked to visuospatial and cognition. Higher intake of legumes and nuts associated with better global cognition, language, and visuospatial domains. Prudent healthy diet linked to better global cognition in women. WD associated with poorer global and executive function in men.	0.002 * 0.001 * 0.001 * 0.019 * 0.023 * 0.005 *	Age Sex Education	Higher adherence to MD, DASH, and prudent healthy diets, along with increased consumption of legumes and nuts, correlate with better cognitive function in older adults. WD associated with poorer cognitive outcomes in men.
Munoz-Garcia et al., 2020 (Spain) [54]	Longitudinal cohort study Healthy participants with higher education, university graduates **Total** (*n* = 806) **Ethnicity** NR	61 ± 6	Saliva	Spanish Telephone Interview for Cognitive Status	WDP MDP	Higher adherence to WD pattern associated with a greater decline in cognitive function, while higher MD adherence associated with less decline in cognitive function.	0.03 * 0.01 *	Age Sex Education Physical activity Smoking Alcohol consumption BMI	High MDP adherence associated with less cognitive decline over 6 y.
Corley et al., 2020 (UK) [55]	Cross-sectional study Healthy older adults, female (50.3%) **Total** (*n* = 511) **Ethnicity** NR	79.3 ± 0.6	MRI	Composite scores for global cognitive function, visuospatial ability, processing speed, memory, and verbal ability	MD dietary pattern and processed dietary pattern.	Higher MD adherence associated with better cognitive function. **Specific associations:** Verbal ability global cognitive function, visuospatial ability, memory. Poor cognitive function with WD.	0.002 * 0.043 * 0.019 * 0.029 *	Age Sex Physical activity Alcohol consumption Smoking Diabetes Stroke Hypertension Hyper-cholesterolemia APOE ε4	Adherence to MD associated with better cognitive functioning but not with brain structural integrity.
Wesselman et al., 2021 (Germany) [56]	Cross-sectional German DELCODE study individuals at increased risk for AD **Female** (52%) **Total** (*n* = 389) **Ethnicity** German	**Female** 69.4 ± 5.6	MRI Blood	CERAD Neuropsychological Battery, ADAS-Cog, FCSRT Wechsler Memory Scale SDMT Face Name Test	MD MIND diet dietary patterns	MD associated with better memory and language. MIND diet associated with better memory. Alcoholic beverages associated with better memory, language, executive function, and working memory.	0.003 * 0.017 * 0.046 * <0.001 *	Age Sex Education Energy intake BMI Smoking Physical activity APOE ε4	MD, MIND diets, and alcoholic beverages associated with better cognitive functions.
Moustafa et al., 2022 (USA) [57]	Cohort Study HCHS/SOL SOL-INCA **Female** (57.8%) **Male** (42.2%) **Total** (*n* = 6321) **Adherence** **Low** (*n* = 2112) **Moderate** (*n* = 2795) **High** (*n* = 1414) **Follow-up** 7 y **Ethnicity** Hispanic or Latino	**Adherence** **Low** 55.7 (55.2–56.3) **Moderate** 56.5 (56.0–57.0) **High** 56.1 (55.4–56.9) **Follow-up** NR	Blood	B-SEVLT Sum B-SEVLT Recall word fluency DSST	24 h recalls MD adherence using MDS **Low:** (0–4) **Moderate:** (5–6) **High:** (7–9)	**High vs. low MD adherence-Visit 1 (crude model):** B-SEVLT sum B-SEVLT Recall Global cognition **High MD adherence vs. low MD adherence-Visit 1 (adjusted model):** B-SEVLT sum B-SEVLT Recall Global cognition **High vs. low MD adherence-Visit 2 (crude model):** B-SEVLT sum B-SEVLT Recall Global cognition	NR	Age Sex Education Hypertension Smoking Physical activity BMI	High MD adherence may reduce the risk of cognitive decline and AD among middle-aged and older Hispanic or Latino adults. MD high adherence associated with better cognitive performance and less cognitive decline over 7 y.
Hassan et al., 2018 (UK) [58]	Cohort study MCI and mild dementia patients **Total** (*n* = 26) **Ethnicity** NR	NR	Blood	NR	MD and exercise, mindfulness, and health self-management in 5-week intervention	Improved quality of life, increased MD adherence, and exercise. 21 (84%) made lifestyle changes due to Brainfood intervention.	0.004 * 0.002 * 0.014 *	NR	Brainfood intervention is feasible in promoting meaningful lifestyle changes in MCI and dementia patients.
Mamalaki et al., 2022 (Greece) [59]	Longitudinal Cohort Study Non-demented community-dwelling older adults Women (60%) **Total** (*n* = 1018) **Ethnicity** Greek	73.1 ± 5.0	NR	Global cognition score, comprehensive neuropsychological battery	TLI MD adherence, sleep duration, physical activity, and engagement in activities of daily living	Higher TLI associated with lower risk of developing dementia. Higher MD adherence, moderate sleep duration, physical activity, and engagement associated with slower decline in cognitive function and developing dementia.	<0.001 * <0.001 * 0.005 * 0.004 * 0.018 * 0.021 * 0.007 * 0.006 * 0.003 * 0.002 *	Age Sex Education	Greater adherence to TLI associated with slower decline in Global cognition score.
Ahn et al., 2022 (UK) [60]	Population-based cross-sectional study, Participants from health and retirement study **Total** (*n* = 3463) **Ethnicity** Caucasian	68.0 ± 10.0	Blood	Global cognition and odds of cognitive decline	High-intensity PA and the MIND diet	No cognitive outcomes for PA+/MIND vs. PA−/MIND−. PA−/MIND+ associated with better global cognition and reduced odds of cognitive decline vs. PA−/MIND−. PA+/MIND+ associated with better global cognition and lower odds of cognitive decline to PA−/MIND.	NS <0.001 * <0.001 * <0.001 * <0.001 *	Age Sex Education Income	Combining high-intensity physical activity and MIND is associated with better cognitive health. PA+/MIND+ was associated with better cognitive health but did not predict decease odds of cognitive decline.
Wengreen et al., 2013 (USA) [61]	Prospective, population-based study Elderly males and females **Demented** (*n* = 355) **Total** (*n* = 5092) **Follow-up** 11 y **Ethnicity** American	73.8 ± 9.9	NR	3MS	142-item FFQ and 24 h recall DASH and MD adherence scores (positive/negative scores)	DASH and MD scores positively correlated in highest quintile of DASH and MD scores. Calcium intake increased in highest vs. lower quantile of DASH. Higher DASH and MD scores associated with higher 3MS scores. Higher intakes of whole grains, nuts, and legumes were associated with higher 3MS scores.	0.001 * NS 0.001 * <0.001 * <0.001 * 0.0054 * <0.0001 * <0.001 * 0.001 * 0.001 *	Age Sex Education BMI Physical activity Smoking Alcohol consumption Multivitamins Smoking Diabetes Stroke Myocardial infarction	Higher DASH and MD adherence associated with higher cognitive function over an 11-year period. Whole grains, nuts, and legumes are likely neuroprotective.
Talegawkar et al., 2012 (Italy) [62]	Prospective, population-based study InCHIANTI study **Total** (*n* = 690) **MDS** ≤3 (*n* = 202) 4–5 (*n* = 299) ≥6 (*n* = 189) **Follow-up** 6 y **Ethnicity** Italian	73.0 ± 6.24	Urine Blood	MMSE	FFQ Adherence to MD (MDS score: ≤3, 4–5, ≥6)	Higher adherence (≥6) associated with lower odds of developing frailty compared with those with lower adherence (score ≤ 3). Higher MD adherence at baseline was associated with a lower risk of low PA and low walking speed.	NR	Age Sex Energy intake Education Smoking BMI MMSE score Presence of chronic diseases	Higher MD adherence inversely associated with development of frailty in older adults, with specific protective effects observed for PA and walking speed.
Tangey, 2014 (USA) [63]	Prospective cohort study Cognitively normal older adults **Total** (*n* = 826) **Ethnicity** NR	81.5 ± 7.1	Blood	19 tests to compute global and summary cognitive scores	FFQ DASH diet (0–10) and MD (MD score) (0–55)	DASH score associated with slower global cognitive decline. 1-unit increase in MD score associated with slower global cognitive decline.	0.03 * 0.01 *	Age Sex Education Cognitive activities Total energy intake Physical activity APOE ε4 status	Higher DASH and MD adherence associated with slower rates of cognitive decline in older adults.
Scarmeas et al., 2009 (USA) [64]	Prospective cohort study Community-dwelling elders without dementia **Total** (*n* = 1880) **Ethnicity** Caucasian, African, Hispanic	77.2 ± 6.6	NR	Standardized neurological and neuropsychological measures	MD-type diet (score 0–9) and physical activity (low, some, much)	Higher MD adherence and physical activity were independently associated with reduced risk of AD. High diet score HR: 0.60 (95% CI: 0.42–0.87), high physical activity HR: 0.67 (95% CI: 0.47–0.95).	0.008 * 0.03 *	Age Sex Education APOE genotype Caloric intake BMI Smoking Comorbidity index	Both higher MD-type diet adherence and physical activity were independently associated with reduced risk for AD.
Calil et al., 2018 (Brazil) [65]	Cross-sectional study Healthy seniors, MCI, and AD individuals **Total** (*n* = 96) **Ethnicity** NR	≥60	NR	BCSB MMSE	MD and MIND diet	Higher MD and MIND adherence associated with better cognitive performance in healthy seniors but not in those with MCI or AD.	0.025 * 0.007 *	Age Education BMI	Moderate MD and MIND adherence associated with better cognition among healthy seniors living in middle–low-income countries, but not those with MCI or AD.
Féart et al., 2011 (France) [66]	Prospective cohort study Healthy elderly community dwellers **Total** (*n* = 1050) **Ethnicity** NR	75.9	Blood	MMSE Isaacs Set Benton Visual Retention Trail Making tests	MD adherence measured by FFQ and 24 h recall, MD score (0–9)	Women with highest MD adherence had a 50% relative risk reduction in incident disability in B-IADL over time compared to those with lowest adherence.	0.01 * 0.003 *	Age Sex Physical activity Smoking BMI APOE genotype	Higher MD adherence associated with favorable plasma fatty acid profiles, a protective effect on cognitive function.
Larsson and Wolk, 2018 (Sweden) [67]	Prospective cohort study **Swedish National Patient Register** (*n* = 28,775) **Dementia patients** (*n* = 3755) **Ethnicity** Swedish	**Baseline:** 71.6 ± 4.5 **Age at diagnosis:** 83.2 ± 5.1	NR	NR	MD DASH diet Smoking Sleep duration Physical activity Alcohol consumption	No significant association between diet, alcohol, coffee consumption, PA, and dementia. **Smoking:** Former smokers vs. never smokers (*n* = 1037 cases). Current smokers vs. never smokers (*n* = 618 cases). **Sleep:** >9 h sleep vs. 7.1–9 h sleep (*n* = 119 cases)**.**	NS 0.006 * 0.045 * 0.001 *	Age Sex Education BMI Hypertension Hyper-cholesterolemia Diabetes Total energy intake	Smoking associated with increased risk of dementia. No evidence that other major lifestyle factors impact risk of late-onset dementia.
Vrijsen et al., 2020 (Netherlands) [68]	Cluster RCT Middle-aged descendants of people with recently diagnosed dementia **Total** (*n* = 378) **Ethnicity** NR	40–60	Blood	Lifestyle for Brain Health score, Motivation to Change Lifestyle and Health Behavior for Dementia Risk Reduction Scale (MCLHB-DRR)	Online tailor-made lifestyle advice on MD	**Primary outcome:** Difference in uptake between active and passive recruitment strategies. **Secondary outcome:** Changes in LIBRA score, individual health behaviors, beliefs and attitudes, compliance to lifestyle advice.	NR	Age Sex Education Socioeconomic	Uptake and effectiveness of online lifestyle program designed to reduce dementia risk factors among middle-aged descendants of people with dementia.
Margara-Escudero et al., 2022 (Spain) [33]	Prospective cohort study Healthy adults aged 30 to 70 years from the EPIC-Spain dementia cohort **Total** (*n* = 25,015) **Follow-up** Mean of 21.5 years **Ethnicity** NR	30–70	NR	Dementia/AD diagnoses confirmed via **First phase:** By linking EPIC-Spain database with health databases containing dementia-related clinical information. **Second phase:** Validated by neurologists based on clinical evaluations	Egg consumption estimated at baseline using a validated dietary history questionnaire Categorized by quartiles (Q1–Q4) Association analyzed with adherence to the rMED score	No overall association between egg consumption and dementia risk. No significant association between egg consumption and AD risk. **Subgroup analysis:** Participants with low adherence to the rMED score, a borderline inverse association was observed.	NR	Age Sex Education Smoking Physical activity BMI Alcohol intake Diabetes Hypertension Stroke Ischemic heart disease Cancer Total energy intake rMED score	Higher egg consumption associated with lower risk of dementia and AD in individuals with low adherence to MD, suggesting that eggs might be a key source of neuroprotective nutrients.
Scarmeas et al., 2006 (USA) [69]	Case–control study **AD** (*n* = 194) **Non-demented** (*n* = 1790) from a community-based cohort in New York **Total** (*n* = 1984) **Ethnicity** NR	**AD:** 82.3 ± 7.5 **Non-AD:** 75.6 ± 6.1	Blood	Memory, orientation, abstract reasoning, language, construction	MD adherence (0–9 scores), high scores = high adherence	Higher MD adherence associated with lower AD risk (OR: 0.76; 95% CI: 0.67–0.87). Participants in lowest tertile vs. middle MD tertile (OR: 0.47; 95% CI: 0.29–0.76) and those at the highest tertile (OR: 0.32; 95% CI: 0.17–0.59) for AD. Vascular variables did not change the magnitude of the association.	<0.001 * <0.001 *	Age Sex Education APOE genotype Smoking BMI Hypertension	Higher MD adherence associated with reduced AD risk independent of vascular comorbidities.
Gardener et al., 2012 (Australia) [70]	Cross-sectional study **Healthy controls** (*n* = 723) **MCI** (*n* = 98) **AD** (*n* = 149) **Total** (*n* = 970) **Ethnicity** Australian	**All:** 71.72 ± 7.86 **AD:** 77.50 ± 8.20 **MCI:** 76.01 ± 7.78 **HC:** 69.94 ± 6.95	Blood	MMSE Logical Memory II CVLT-II D-KEFS-VF	MD adherence	Higher MD adherence associated with lower risk of AD MCI. Significant correlation between baseline MD score and change in MMSE score over 18 months.	<0.001 * 0.04 *	Age Sex Education APOE genotype Smoking BMI Diabetes Hypertension	Higher adherence to MD is associated with reduced risk of AD and MCI independent of vascular comorbidities.
Vlachos et al., 2021 (Greece) [71]	Prospective cohort study Cognitively normal elderly individuals who reported SCD decline **Total** (*n* = 939) **Ethnicity** Greek	74.1 ± 6.3	Peripheral blood	Structured questionnaires assessing SCD in memory, language, visuoperceptual, and executive domains	MDS	Higher MDS by 10 points associated with 7% reduction in SCD progression within 1 year. Each additional vegetable serving per day associated with 2.2% reduction in SCD progression.	0.02 * 0.03 *	Sex Age Education APOE-ϵ4 status	Higher MD adherence associated with slower SCD progression. Increased vegetable consumption is beneficial.
Rocaspana-García et al., 2018 (Spain) [72]	Cross-sectional study AD patients living at home **Total** (*n* = 111) **Ethnicity** Spanish	78.5 ± 6.4	Blood	MMSE GDS NPI	Nutritional data using MNA, FFQ	Nutritional status correlated with MMSE. Inverse correlation with functional status, NPI, and ZBI scales.	0.001 * 0.004 *	Age Sex Family history Hypertension Diabetes Dyslipidemia Depression BMI	Low MD adherence associated with worse cognitive, functional, and behavioral outcomes.
Gu et al., 2010 (USA) [73]	Prospective study **AD cases** (*n* = 118) **Non-demented elderly** **Total** (*n* = 1219) **Follow-up** 4 y **Ethnicity** NR	78.5 ± 6.4	Blood	Neuropsychological battery AD diagnosis using NINCDS-ADRDA criteria	61-item SFFQ for MD adherence	Better MD adherence associated with lower hsCRP but not with fasting insulin and adiponectin. Higher MD adherence associated with 34% lower AD risk.	0.003 * 0.04 *	Age Sex Education APOE genotype BMI Smoking Caloric intake Ethnicity Comorbidities	Lower risk of AD with better MD.
Hu et al., 2020 (USA) [74]	Prospective cohort study Healthy participants from ARIC study **Total** (*n* = 13,630) **Ethnicity** NR	45–64	NR	Incident dementia diagnosed via neurocognitive battery, informant interviews, hospitalization, or death codes	HEI-2015 AHEI-2010 aMeD DASH scores	Higher adherence to HEI-2015 associated with a lower risk (14%) of developing dementia comparing highest to lowest quantile. No significant associations for AHEI, aMed, and DASH scores.	0.01 * NS	Age Sex Education APOE ε4 genotype Smoking Physical activity Alcohol consumption BMI	Adherence to HEI-2015 during midlife was associated with lower risk of dementia.
Tanaka et al., 2018 (Italy) [75]	Longitudinal cohort study Participants without CI at baseline **Total** (*n* = 832) **Ethnicity** Italian	75.4 ± 7.6	Blood	MMSE	MDS categorized into low (≤3), mild (4–5), and high (≥6) adherence	High MD adherence decreased likelihood of cognitive decline MD adherence was protective.	<0.001 * 0.03 *	Age Sex Education Physical activity Smoking BMI APOE ε4 genotype	MD adherence protective against cognitive decline over 18-year period. Protective effect is strongest in those with high adherence.
Scarmeas et al., 2007 (USA) [76]	Longitudinal cohort study Community-based AD individuals **Total** (*n* = 192) **Follow-up** Every 1.5 y **Ethnicity** Americans	**Baseline** 82.9 ± 7.7	NR	Neuropsychological battery including memory, orientation, abstract reasoning, language, and construction tests	FFQ	Higher MD adherence associated with significantly lower mortality risk. MD score was associated with a 24% lower risk of death in adjusted models.	0.001 * 0.003 *	Age Sex Education APOE genotype Smoking BMI Caloric intake Ethnicity Recruitment period	MD adherence associated with lower mortality in AD patients, suggesting a dose–response effect. Higher adherence leads to longer survival in AD patients.
Vu et al., 2022 (USA) [77]	Cohort study **CHAP** (*n* = 2449) **MAP** (*n* = 725) **WHIMS** (*n* = 5308) **Ethnicity** **(CHAP)** Mixed **(MAP)** Non-Hispanic Caucasian European ancestry	**MAP:** 78.5 **WHIMS:** 86 **CHAP:** 79	NR	**CHAP and MAP:** Battery of cognitive tests **WHIMS:** Screened annually with the 3MSE	MIND diet adherence score (15 food items)	**MAP and WHIMS:** Higher MIND adherence associated with lower dementia risk and slower cognitive decline. **CHAP:** No association.	<0.02 * NS	Age Sex Education	Genetic risk and MIND adherence are independently associated with dementia risk. No gene–diet interaction replicated across cohorts.
Klinedinst et al., 2020 (UK) [34]	Longitudinal cohort Mid-to-late-aged adult UK Biobank participants **Total** (*n* = 1787) **Ethnicity** NR	63 ± 7.4	Blood	FIT over 10 years	Daily cheese, alcohol (type and frequency), lamb, red wine, added salt	Daily cheese intake positively associated with cognitive scores over time. **Genetic risk:** Daily alcohol beneficial Salt correlated with decreased cognitive performance **Family history:** Red wine protective. Weekly lamb consumption improved cognitive outcomes.	<0.001 * 0.022 * 0.014 * 0.008 * 0.004 *	Age Sex Education Socioeconomic BMI Smoking	Cheese and red wine daily and lamb weekly may improve long-term cognitive outcomes. High salt intake should be minimized, especially for those at genetic risk.
Titova et al., 2013 (Sweden) [78]	Longitudinal cohort study **PIVUS cohort:** Cognitively healthy older individuals **Total** (*n* = 194) **Ethnicity** Swedish	70	MRI	7MS	MD based on high intake of fruits and low intake of meat	Low meat intake associated with better performance on cognitive tests and greater brain volumes.	0.001 * 0.03 *	Sex Energy intake Education Physical activity BMI	Low meat intake associated with better cognitive performance and greater brain volumes in elderly.
Hoscheidt et al., 2022 (USA) [79]	RCT Middle-aged adults with **Normal cognition** (NC) (*n* = 56) **MCI** (*n* = 31) **Total** (*n* = 87) **Ethnicity** NR	**MCI:** 56.2 ± 5.1 **NC:** 56.3 ± 5.1	CSF Blood MRI	3MS	Western-like/WD vs. MD-like/MD for 4 weeks. Isocaloric diets **High** SF, high GI, high Na+ **Low** SF, low GI, low Na+. **MD** (*n* = 44) **WD** (*n* = 43)	**WD and MD in NC and MCI:** Reduced total cholesterol. LDL and HDL Fasting plasma glucose and insulin **Diet groups and AD biomarkers:** Decreased Aβ40 with MD and increased after WD. **NC group:** Increased ratios with MD and reduced after WD, but MCI had reverse pattern. Diet-affected t-tau levels for MCI increased by MD and decreased by WD. MCI showed reduced CSF Aβ42/t-tau ratios with MD and increased with WD. Ratios unchanged for NC. **Cerebral perfusion:** Significant in left inferior frontal cortex, right middle temporal gyrus, and para-hippocampal gyri for MD > WD.	0.0001 * 0.0001 * 0.0006 * 0.007 * 0.012 * 0.049 * 0.014 * NS 0.038 * 0.044 * 0.036 * NS 0.003 * NS	Age Sex BMI Baseline 3MS scores APOE ε4 status Test site	Diets modulate AD biomarkers, cerebral perfusion, and cognition differently for NC and MCI adults. MD benefits NC, West-diet benefits some markers in MCI. Perfusion increased following MD and decreased following WD for the NC group. MCI group perfusion levels increased following MD.
Ye et al., 2013 (USA) [80]	Cross-sectional study Middle-aged and older adults, generally healthy **Total** (*n* = 1269) **Ethnicity** Puerto Rican	57.3 ± 7.6	NR	MMSE	MD adherence (0–9 scale) and HEI 2005 score	Higher MD adherence associated with better global cognitive function and lower likelihood of cognitive impairment. Higher HEI 2005 score associated with better global cognitive function and lower odds of cognitive impairment.	0.012 * 0.001 * 0.011 * 0.033 *	Age Sex Education Smoking Physical activity BMI	High adherence to MD or HEI 2005 protects cognitive function in middle-aged and older adults.
Roberts et al., 2010 (USA) [81]	Longitudinal cohort study **Non-demented** (*n* = 1233) **MCI** (*n* = 163) **Ethnicity** NR	70–89	Blood	CDR Functional Activities Questionnaire Short Test of Mental	MD adherence with 128-item FFQ	**MCI cases:** Lower vegetable intake MUFA + SUFA ratio moderate alcohol consumption but higher caloric intake vs. controls. Higher legume, grain, cereals, and red meat and moderate alcohol consumption but lower fruit and vegetable intake in men vs. women. **MCI men:** Lower fish intake but higher caloric intake. OR of MCI decreased significantly with higher vegetable intake. OR (95% CI) for the middle and upper tertiles compared to the lowest tertile: Vegetable intake Moderate alcohol (MUFA + PUFA) SFA ratio.	0.06 0.05 0.05 0.0005 * 0.014 * 0.003 * <0.001 * <0.01 * 0.004 * 0.0001 * 0.005 * 0.05 0.001 * 0.04 * 0.003 * 0.008 *	Age Education Sex Stroke ApoE ε4 status Coronary heart disease Depression MCI status	Vegetables, unsaturated fats, and a high MD score may be beneficial to cognitive function. Risk of incident MCI or dementia was reduced in subjects with a high MeDi score. High vegetable intake and unsaturated fats reduced odds of MCI. High MD score reduced risk of dementia.
Scarmeas et al., 2009 (USA) [82]	Prospective cohort study **Cognitively healthy** (*n* = 1393) **MCI** (*n* = 275) **Total** (*n* = 1668) **Follow-up** NR **Ethnicity** Multiethnic (Caucasian, African, Hispanic)	**Baseline** 76.9 ± 6.5	Blood	Neuropsychological battery CDR	MD adherence (0–9 scale) Low/moderate and high MD adherence	Moderate MD tertile less risk of developing MCI vs. low MD tertile. Higher MD adherence less risk of developing MCI and AD. Moderate MD adherence 45% less chance of developing AD compared to low MD adherence subjects.	**0.24** **0.05** 0.01 * **0.02**	Age Sex Education APOE genotype Caloric intake BMI Time between baseline dietary assessment and baseline diagnosis	Higher MD adherence associated with reduced risk of developing MCI and progression from MCI to AD, suggesting a protective effect of the diet.
Trichopoulou et al., 2014 (Greece) [83]	Prospective cohort study Healthy elderly men and women **Total** (*n* = 401) **Ethnicity** Greek	≥65	NR	MMSE	MDS: 0–3 low, 4–5 interMDate, 6–9 high	Higher MD adherence associated with lower cognitive decline. **OR for mild vs. no decline:** 0.46 (95% CI: 0.25–0.87). **OR for substantial vs. no decline:** 0.34 (95% CI: 0.13–0.89).	0.012 * 0.025 *	Age Sex Education Smoking Physical activity Total energy intake	MD adherence associated with reduced cognitive decline, with higher vegetable consumption playing a key role.
Dobreva et al., 2022 (UK) [84]	Cohort study Participants from UK Biobank **Total** (*n* = 249,511) **Ethnicity** NR	62 ± 4	NR	Incident dementia ascertained through electronic health records	MD components: Fruit, vegetables, processed meat, unprocessed red meat, unprocessed poultry, fish, cheese, wholegrains	Moderate fish consumption associated with decreased dementia risk. Fruit consumption associated with reduced dementia risk.	0.034 * 0.040 *	Age Sex Socioeconomic Education Physical activity Smoking Alcohol consumption BMI Hypertension Dietary changes	Fish consumption may drive beneficial effects of MD on dementia risk. Need to establish mechanisms and potential interventions.
Soldevila-Domenech et al., 2024 (Spain) [85]	RCT PREDIMET-Plus Cognition sub-study APOE-ε4 carriers **Women** (53.9%) **Men** (46.1%) **APOEε4 carriers** (18.8%) **Total** (*n* = 102) **Follow-up** 3 y **Ethnicity** Spanish	**Baseline** 65.6 ± 4.5 **Follow-up** **1 year** 62.1–71.1 **3 year** 64.1–73.1	Blood	Executive function Global cognition Memory	14 items MEDAS questionnaire. MD intervention over 3 years, evaluation of changes in plasma concentrations of eCBs: 2-AG, AEA, OEA, PEA, DHEA	**Men:** Better executive function and global cognition vs. women but non-significant in sex differences models. **3 years MD intervention:** Improvement in memory and global cognition. Greater memory changes in men vs. women. OEA increase positively associated with memory performance. OEA/AEA ratio positively associated with memory in men vs. women. **After 1 year:** Increase in OEA/PEA associated with memory improvements in men. **After 3 years:** Increase in DHEA concentrations in women linearly associated with global cognition improvements. **After 6 months of MD intervention:** 2-AG, AEA, OEA, PEA, DHEA, DGLEA, LEA, POEA, and SEA decreased in APOEε4 noncarriers remained unchanged in APOE-ε4 carriers.	0.036 * 0.017 * 0.049 * 0.067 0.062 0.026 * 0.062 0.0034 * 0.042 * 0.064 0.012 * 0.043 * 0.009 * 0.009 * 0.003 * 0.007 * 0.006 * 0.048 * 0.031 * 0.051 *	Sex APOE genotype Metabolic syndrome Baseline cognition Lipid metabolism markers	MD improved cognitive performance modestly, with greater benefits observed in men. Cognitive changes associated with endocannabinoid system modulation in a sex- and APOE-dependent manner. Supports eCB system modulation as a potential therapeutic approach to prevent cognitive decline in at-risk populations.
McGrattan, 2021 (UK) [86]	Pilot RCT **MCI** (*n* = 15) **SCI** (*n* = 5) **Total** (*n* = 20) **Ethnicity** North Irish	**MCI** 76.5 ± 7.0 **SCI** 67.2 ± 7.9	Blood	CANTAB cognitive test battery	**MD components:** Fruits, vegetables, olive oil, oily fish, nuts, whole grains	Improved cognition with MD intervention groups at 6 months.	NR	Age Sex Education Baseline diet Cognitive status	MCI patient recruitment was difficult. Community-based recruitment from SCI participants showed better feasibility.
Shannon et al., 2019 (UK) [87]	Prospective cohort study **EPIC-Norfolk study group:** Older adults **Total** (*n* = 8009) **Ethnicity** British	55	Blood	SF-EMSE HVLT VST CANTAB-PAL	MD adherence (Pyramid and MEDAS scores)	**Higher MD adherence associated with the following:** Better global cognition, verbal episodic memory. Lower risk of poor verbal episodic memory. Better processing speed. Lower risk of poor complex processing speed and prospective memory. **High CVD risk individuals:** Lower risk of poor verbal episodic memory and complex processing speed associated with better global cognition, verbal episodic memory.	0.018 * 0.008 * <0.001 * <0.001 * 0.026 * 0.018 * 0.013 * 0.004 * 0.045 * 0.029 * 0.023 * 0.021 * 0.015 * 0.007 * 0.003 *	Age Sex BMI Waist circumference Marital status Employment status Cholesterol Triglycerides Smoking Physical activity Education APOE genotype	Higher MD adherence associated with better cognitive function and lower risk of poor cognition in older UK adults, especially in those with higher CVD risk.
Anastasiou et al., 2017 (Greece) [88]	Population-based cohort study Healthy and cognitively impaired older adults **Total** (*n* = 1865) **Ethnicity** Greek	73 ± 6	NR	Memory, language, attention-speed, executive functioning, visuospatial perception, composite cognitive score	MD adherence	Each unit increase in MD score associated with 10% decrease in dementia. Better performance in memory, language, visuospatial perception, and cognitive score. Fish consumption negatively associated with dementia.	<0.001 *	Age Sex Education ApoE genotype	Higher MD adherence associated with better cognitive performance and lower dementia rates. Non-refined cereals and fish consumption also beneficial.
Gu et al., 2015 (USA) [89]	Cross-sectional study Elderly adults without dementia **Total** (*n* = 674) **Ethnicity** Multiethnic (African American, Hispanic, Caucasian non-Hispanic)	80.1 ± 5.6	MRI	Neuropsychological battery (A range of standardized tests to assess various cognitive domains)	MD score 0–9 **Low adherence** (0–4) **High adherence** (5–9)	Higher MD adherence associated with larger total brain volume, gray matter, and white matter volume. Higher fish intake associated with larger total gray matter volume and mean cortical thickness. Lower meat intake associated with larger total gray matter and brain volume.	0.007 * 0.006 * 0.002 * 0.02 * 0.03 *	Age Sex Ethnicity Education BMI Diabetes Cognition	Higher MD adherence associated with less brain atrophy. Fish and lower meat intake are key contributors.
Nicoli et al., 2021 (Italy) [90]	Population- based cohort study Monzino-80 plus study in cross-sectional study of older adults **Total** (*n* = 1390) in longitudinal study **Total** (*n* = 512) **Ethnicity** Caucasian	**Cross-sectional study** 92 ± 5.3 **Longitudinal study** 94 ± 5.3	Blood	MMSE DSM-IV criteria	FFQ MD score, consumption of specific food groups (eggs, fruits, vegetables, carbohydrates, legumes) and overall food intake	Legumes consumption associated with decreased dementia incidence. Greater quantity of food intake associated with lower dementia incidence. Higher intake of fruits/vegetables, carbohydrates, and eggs associated with lower dementia prevalence. **Univariable model (Model 1)** MDS, fruits and vegetables, meat, bread, rice and pasta, milk and cheese, eggs, legumes, fish, sweets, variety and quantity in food intake **Partially adjusted model (Model 2)** MDS, fruits and vegetables, meat, bread, rice and pasta, milk and cheese, eggs, legumes, fish, sweets, variety and quantity in food intake **Fully adjusted model (Model 3)** MDS, fruits and vegetables, meat, bread, rice and pasta, milk and cheese, eggs, legumes, fish, sweets, variety and quantity in food intake.	0.008 * 0.038 * 0.027 * <0.001 * <0.001 * <0.001 * <0.001 * <0.001 * <0.001 * <0.001 * 0.039 * <0.001 * <0.001 * NS <0.001 * <0.001 * <0.001 * 0.002 * <0.001 * NS <0.001 * NS <0.001 * 0.036 * NS 0.001 * <0.001 * <0.001 * 0.008 * <0.001 * NS <0.001 * NS <0.001 * NS NS NS 0.004 * <0.001 *	Age Sex Education Total caloric intake Smoking Alcohol consumption Physical activity Hypertension Diabetes Depression	Higher MD adherence and specific food consumption patterns, linked to lower dementia prevalence and incidence. High MD adherence greater consumption of eggs, fruits and vegetables, carbohydrates, and greater food intake were associated with a lower prevalence of dementia. Increasing number of portions per week and consumption of legumes significantly decreased the incidence of dementia during the 3.6 year mean follow-up.
Filippini et al., 2020 (Italy) [91]	Case–control study Newly diagnosed EOD patients **Total** (*n* = 54) **Ethnicity** NR	66	NR	EOD diagnosis based on clinical criteria	FFQ Greek-MD, DASH, and MIND diets adherence	Cereal intake showed a U-shaped relation with EOD risk. High dairy intake (>400 g/day) associated with increased risk. Inverse relation with vegetable intake (>100 g/day), citrus, and dry fruits.	0.032 * 0.041 * 0.038 *	Sex Age Education Total energy intake	High intake of cereals and dairy associated with increased EOD risk. Higher vegetable, citrus, and dry fruit intake and adherence to the MIND diet associated with decreased EOD risk.
Crom et al., 2022 (Netherlands) [92]	Population-based cohort study **Baseline I:** Healthy adults aged 55 years and older (*n* = 5375) **Baseline II:** Healthy adults turned 55 years or moved to study area (*n* = 2861) **Total** (*n* = 8236) **Dementia** **Total** (*n* = 1188) **Dementia at follow-up** (*n* = 248) developed dementia at 5.9 years follow-up **Follow-up** 3–5 y **Ethnicity** NR	**Baseline I:** 67.7 ± 7.8 **Baseline II:** 75.3 ± 5.9	NR	MMSE GMS	FFQ MIND diet, Dutch dietary guidelines, MD	**Baseline I:** MIND diet score not associated with dementia risk (model 2 adjusted HR [95% CI] per SD increase, 1.00 [0.94, 1.06]). **Baseline II:** Higher MIND diet score associated with a lower dementia risk over every follow-up, but associations slightly attenuated over time (HR [95% CI] for 7 years follow-up per SD increase, 0.76 [0.66, 0.87]).	0.029 * <0.001 *	Age Sex Education Smoking Physical activity Energy intake BMI Diabetes Hyper-cholesterolemia Hypertension Depression APOE genotype	MIND diet adherence is associated with decreased dementia risk, particularly in the short term. The MIND diet score at baseline II was more strongly associated with the risk of dementia than the MIND diet score at baseline I.
Vassilaki et al., 2018 (USA) [93]	Cross-sectional study Cognitively unimpaired older adults of Northern European ancestry **Total** (*n* = 278) **Ethnicity** Northern European	77.7 ± 7.9	PET	NR	MD score, consumption of specific food groups, including vegetables, legumes, fish, grains, fruit, red meat, dairy, alcohol	Higher MD adherence associated with lower Aβ deposition. Higher vegetable intake, vitamin A, β-carotene intake, and moderate alcohol consumption linked to lower Aβ levels.	0.012 * 0.002 * 0.03 * 0.003 * 0.005 *	Age Sex Education APOE ε4 status Time interval between FFQ completion and PET scan Total energy intake BMI	Higher MD adherence to specific dietary components associated with lower Aβ deposition, suggesting a potential protective effect of the diet against AD.
Feart et al., 2009 (France) [94]	Prospective cohort study **Three city cohort:** Elderly persons **Total** (*n* = 1410) **Dementia** (*n* = 99) **Follow-up** 5 y **Ethnicity** French	75.9 ± 4.8	NR	MMSE IST BVRT FCSRT	MD score (0–9) based on FFQ and 24 h recall	Higher MD adherence associated with fewer MMSE errors. FCSRT, IST, or BVRT performance over time was not significantly associated with MD adherence. Association of greater MD adherence with fewer MMSE errors and better FCSRT scores not significant in entire cohort, but those who were dementia-free for over 5 years, association was significant (adjusted for all factors except stroke, MMSE, and FCSRT).	0.03 * NS NS 0.03 * 0.03 * 0.53	Age Sex Education Marital status Energy intake Physical activity Depression ApoE genotype	Higher MD adherence associated with slower MMSE decline but not with other cognitive tests. MD adherence not associated with incident risk for dementia.
Bhushan et al., 2017 (USA) [95]	Prospective cohort study Male health professionals **Total** (*n* = 27,842) **Ethnicity:** Caucasian	51	NR	Self-reported SCF assessed by 6-item questionnaire	MD score computed from FFQs (mean of five FFQs assessed every 4 years from 1986 to 2002)	Men in highest quantile of MD adherence had 36% lower odds of poor SCF (OR: 0.64, 95% CI: 0.55–0.75) and 24% lower odds of moderate SCF.	<0.001 * <0.001 *	Age Smoking Diabetes Hypertension Depression Cholesterol Physical activity BMI	Long-term MD adherence associated with better subjective cognitive function in later life.
McMaster et al., 2018 (Australia) [96]	RCT Community-dwelling individuals with MCI or SCD **Total** (*n* = 160) **Follow-up** 3–6 months **Ethnicity** NR	≥65	Blood	ADAS-Cog-Plus ANU-ADRI	8-week **Multidomain lifestyle intervention:** MD, PA, cognitive engagement **Control group:** Dementia literacy Lifestyle risk MD, PA Cognitive engagement **Intervention group:** Dementia literacy Lifestyle risk MD, PA Cognitive engagement Dietitian session Online brain training Exercise Physiologist	Higher adherence to intervention associated with improved cognitive scores and reduced lifestyle risk factors for AD.	0.045 * 0.022 *	Age Sex Baseline ADAS-Cog-Plus Baseline ANU-ADRI Adherence to intervention components	Multidomain lifestyle interventions could reduce cognitive decline and AD risk in elderly individuals with SCD or MCI.
Dhana et al., 2022 (USA) [97]	Prospective cohort study Participants categorized based on adherence to healthy lifestyle factors: **0–1 healthy factors:** Women (*n* = 411) Men (*n* = 215) **2–3 healthy factors:** Women (*n* = 878) Men (*n* = 536) **4–5 healthy factors:** Women (*n* = 251) Men (*n* = 158) **Total** (*n* = 2449) **Ethnicity** African American	76 ± 6.8	NR	Structured clinical neurological evaluations with neuropsychological testing	**Five modifiable lifestyle factors:** MIND diet, cognitive activities, physical activity, no smoking, and moderate alcohol consumption	Adherence to 4–5 healthy lifestyle factors significantly reduces risk of developing AD/dementia in both women and men. Adherence to 4–5 healthy lifestyle factors significantly reduces the risk of mortality in both women and men.	<0.001 * <0.001 * <0.001 * <0.001 * 0.028 *	Age Race Marital status Education APOE ε4 status	A healthy lifestyle is associated with longer life expectancy and fewer years lived with AD or dementia.
Puente-González et al., 2020 (Spain) [98]	RCT Institutionalized AD patients **Total** (*n* = 84) **Ethnicity** NR	≥50	NR	MMSE GDS	MPEP combined with MD. **Nutritional status:** MNA will evaluate nutritional status of patients	MD expected to improve bone mass due to high nutrient content. **Gait and balance:** Improved nutritional status from the MD, rich in antioxidants and anti-inflammatory components, expected to enhance gait and balance. **Fall risk:** Improve bone health and physical function, the MD, in conjunction with exercise, aim to reduce overall risk of falls.	NR	Age Sex Baseline physical activity	Multicomponent physical exercise program with MD significantly improves gait, balance, and bone health, thereby reducing fall risk in patients. Better nutritional status is linked to overall health improvements and potentially slower progression of AD symptoms.
Ntanasi et al., 2017 (Greece) [99]	Cross-sectional study Greek older adults **Total** (*n* = 1740) **Ethnicity** Greek	73.4 ± 5.4	NR	Comprehensive neuropsychological assessment	MD adherence (MD score)	Frailty measured by three definitions: Fried, Frailty Index, and Tilburg Frailty Indicator. Higher adherence to MD associated with lower odds of frailty.	0.023 * 0.005 * 0.031 * <0.001 *	Age Sex Education	Higher MD adherence linked to lower frailty odds, irrespective of the definition used.
Karstens et al., 2019 (USA) [100]	Cross-sectional study Community-dwelling older adults **Total** (*n* = 82) **Ethnicity** NR	68.8 ± 6.8	MRI	California Verbal Learning Test Trail Making Test Wechsler Adult Intelligence Scale MMSE	MD adherence measured via Block 2005 FFQ	Higher MD adherence associated with better learning and memory performance and larger dentate gyrus volumes.	0.01 * 0.03 *	Age Sex Education BMI	Higher MD adherence associated with better cognitive performance in learning and memory and larger dentate gyrus volumes in healthy older adults.
Enrique de la Rubia Ortí, 2018 (Spain) [101]	Prospective, longitudinal, qualitative, analytic, experimental study AD patients (*n* = 44) **Ethnicity** NR	65–85 years	NR	7-MS (Benton’s temporal orientation test), CDT Categorical Verbal Fluency test Free and Cued Selective Reminding Test	Coconut oil-enriched MD	Improved episodic, temporal orientation, and semantic memory. Differences observed based on sex and severity. Interaction “Time” × “Group” significant for temporal orientation. Interaction “Time × Sex × Group × State” significant for semantic memory.	0.024 * 0.032 * <0.001 *	Sex Disease stage (mild/moderate vs. severe) Education	Coconut oil-enriched MD appears to improve cognitive functions in AD patients, with more pronounced effects in women and those in the mild to moderate disease stages.
Gu et al., 2021 (USA) [102]	Cross-sectional study National Health and Nutrition Examination Survey (NHANES) 2011–2014 Healthy older adults **Total** (*n* = 2435) **Tertiles of MD** **T1 (low)** (*n* = 841) **T2 (moderate)** (*n* = 1129) **T3 (high)** (*n* = 465) **Ethnicity** Hispanic, African American, Caucasian	**Total** 68.99 (6.58) **T1** 68.02 (6.45) **T2** 69.45 (6.60) **T3** 69.57 (6.62)	NR	CERAD-IR CERAD-DR AF DSST	24 h dietary recall interview and MD adherence assessed by a 9-component score. **Tertiles of MD** **T1** (0–3) **T2** (4–5) **T3** (6–9)	**MD** Overall T1 vs. T2 T1 vs. T3 T2 vs. T3. **Dietary calories** Overall T1 vs. T2 T1 vs. T3 T2 vs. T3. **MD and cognitive Scores:** **Moderate MD** (4–5) Global cognition CERAD-IR CERAD-DR AF DSST **High MD** (6–9) Global cognition CERAD-IR CERAD-DR AF DSST.	<0.001 * <0.001 * <0.001 * <0.001 * <0.001 * <0.001 * 0.012 * 0.006 * 0.013 **0.001** NS 0.031 * 0.004 * 0.035 * 0.040 * NS 0.006	Age Sex Race/ethnicity Education Marital status PIR Smoking BMI Physical activity Depression Dietary calories	High adherence to MD associated with better cognition, with effects stronger in non-Hispanic Whites and men. Prospective studies with diverse samples needed to confirm findings. Higher MD score associated with better global cognitive function.
Dhana et al., 2020 (USA) [103]	Prospective cohort study **CHAP** (*n* = 1845) **MAP** (*n* = 920) **Total** (*n* = 2765) **Ethnicity** NR	**CHAP** 73.2 ± 5.8 **MAP** 81.1 ± 7.2	Blood	NINCDS-ADRDA Evaluations every 3 years in a stratified random sample	Healthy lifestyle score based on non-smoking, physical activity, alcohol consumption, diet quality, and cognitive activities	Lower risk of AD/dementia with increased healthy lifestyle behaviors.	<0.001 *	Age Sex Race Education APOE ε4 status	A healthy lifestyle composite score associated with substantially lower risk of AD/dementia.
Morris et al., 2015 (USA) [26]	Prospective cohort study MAP study participants **Total** (*n* = 923) **AD cases** (*n* = 144) **Follow-up** 4.5 years **Ethnicity** NR	81.0 ± 7.0	Blood	Annual neurological exams, structured cognitive tests	SFFQ to assess MIND, DASH, and MD	Adjusted proportional hazards models, second HR = 0.65, 95% CI: 0.44–0.98) and highest tertiles (HR = 0.47, 95% CI 0.26–0.76) of MIND diet scores had lower rates of AD versus tertile 1. Third tertiles of DASH (HR = 0.61, 95% CI 0.38–0.97) and MD (HR = 0.46, 95% CI: 0.26–0.79) associated with lower AD rates.	0.006 * 0.004 * 0.01 * 0.08 0.04 *	Age Sex Education APOE-ε4 Physical activity Depression BMI	DASH and MDs showed protective effects only at the highest adherence levels. 53% reduction in AD those in highest tertile of MIND scores and a 35% reduction for middle tertile scores compared with the lowest tertile.
Liu et al., 2021 (USA) [104]	RCT Overweight individuals with suboptimal diets at AD risk **Total** (*n* = 604) **Ethnicity** Caucasian (88%), African American (11%), other (1.3%)	70.4 ± 4.2	Blood Urine MRI	MoCA, neuropsychological test battery at baseline, 6, 12, 24, and 36 months	3-year intervention period. MIND diet with mild caloric restriction vs. usual diet with mild caloric restriction (250 kcal/d)	MIND diet showed significant reduction in the rate of cognitive decline, significant improvements in brain structure (total brain volume and hippocampal volume).	NR	Age Sex Education BMI Caloric intake Physical activity APOE-ε4 status	MIND diet reduces the rate of cognitive decline and total brain volume loss in older adults at risk for AD.
Ballarini et al., 2021 (Germany) [105]	Cross-sectional study **Cognitively normal** (*n* = 162) **Relatives with AD** (*n* = 53) **SCD** (*n* = 209) **MCI** (*n* = 81) **Total** (*n* = 512) **Ethnicity** German	69.5 ± 5.9	CSF MRI	Extensive neuropsychological battery	MD adherence	Larger temporal gray matter volume, better memory, less amyloid and tau pathology associated with higher MD adherence.	0.038 * 0.008 * 0.004 *	Age Sex Education BMII Caloric intake Physical activity APOE-ε4 status	MD is a protective factor against memory decline and temporal atrophy, possibly by decreasing amyloidosis and tau pathology.
Scarmeas et al., 2006 (USA) [106]	Prospective cohort study Non-demented individuals at baseline, **Total** (*n* = 2258) **AD cases during study** (*n* = 262) **Follow-up** 4 y **Ethnicity** Multiethnic	**Non-demented** 76.5 ± 6.3 years **AD cases** 81.8 ± 6.9 years	Blood	CDR	MD score (0–9 scale)	**AD with higher MD adherence:** HR: 0.91 (95% CI: 0.83–0.98) per unit increase in MD score, 0.60 (95% CI: 0.42–0.87) for highest tertile vs. lowest tertile. Middle MeDi tertile HR: 0.85 (95% CI: 0.63–1.16) vs. highest tertile, HR: 0.60 (95% CI: confidence 0.42–0.87) for AD.	0.015 * 0.007 *	Age Sex Ethnicity Education APOE genotype Smoking BMI	Higher MD adherence associated with lower risk of AD.
Berti et al., 2018 (USA) [107]	Longitudinal cohort study Healthy middle-aged adults **Total** (*n* = 70) **Ethnicity** Mixed	50 ± 8	PET MRI	MMSE	MD score	**Low MD adherence:** Reduced FDG-PET glucose metabolism and higher PiB-PET deposition in AD-affected regions. Low MD adherence showed CMRglc declines and PiB increases compared to those in the high MD adherence group.	<0.001 * <0.001 *	Age Sex Education APOE status BMI	Lower MD adherence associated with progressive AD biomarker abnormalities. 1.5 to 3.5 years protection against AD with higher MD adherence.
Encarnación Andreu-Reinón et al., 2021 (Spain) [108]	Prospective cohort study Participants from EPIC-Spain dementia cohort study **Total** (*n* = 16,160) **Dementia** (67%) (*n* = 459) **Ethnicity** NR	**Baseline** 48.3 ± 12.1 **AD** NR	NR	NR	FFQ and rMED score **Low** ≤6 points **Medium** 7–10 points **High** ≥11 points **Low** (*n* = 3114) **Medium** (*n* = 8163) **High** (*n* = 4424)	High vs. low MD adherence. rMED score associated with 20% lower risk of dementia overall **Baseline** **Non-cases** Energy intake Potatoes, vegetables, fruits, legumes fish and seafood, cereals, olive oil, nuts and seeds, meat, dairy products, eggs, coffee, tea, alcohol rMED score **Baseline** **AD cases** Energy intake Potatoes, vegetables, fruits, legumes, fish and seafood, cereals, olive oil, nuts and seeds, meat, dairy products, eggs, coffee, tea, alcohol rMED score.	NR <0.001 * <0.001 * < 0.001 * <0.001 * <0.001 * <0.001 * <0.001 * <0.001 * <0.001 * <0.001 * <0.001 * <0.001 * <0.001 * <0.001 * <0.001 * NS 0.002 * <0.001 * <0.001 * <0.001 * <0.001 * <0.001 * <0.001 * NS <0.001 * <0.001 * NS NS 0.001 * <0.001 *	Sex Education Smoking BMI Waist circumference Physical activity Hypertension Hyperlipidemia	MD adherence associated with a lower risk of dementia, with stronger associations observed in women for non-AD/dementia and in those with lower educational levels.
Dhana et al., 2021 (USA) [109]	Longitudinal clinical pathological study Community-dwelling older adults **Total** (*n* = 569) **Ethnicity** NR	90.8 ± 6.1	Brain tissue	Episodic, semantic, and working memory, perceptual speed, visuospatial ability	MIND diet score based on FFQ	Higher MIND diet score associated with better global cognitive functioning proximate to death.	0.003 *	Age at death Sex Education APOE ε4 Late-life cognitive activities Total energy intake	MIND is associated with better cognitive functioning independently of brain pathologies, suggesting cognitive resilience.
Nagpal et al., 2019 (USA) [110]	RCT, cross-over, single-center pilot study, **MCI** (*n* = 11) **Cognitively healthy** (*n* = 6) **Total** (*n* = 17) **Ethnicity** American	64.6 ± 6.4	Fecal CSF	Cognitive testing using ADNI-2 criteria for MCI	MMKD vs. AHAD	Enterobacteriaceae positively associated with tau-p181 and tau-p181/Aß42 ratio after MMKD. Bifidobacterium decreased significantly in MMKD. Propionate correlated negatively with Aß42 in MCI after MMKD. Butyrate levels increased after MMKD. Lactate levels decreased in MCI subjects after MMKD.	0.04 * 0.03 * 0.04 * 0.03 * 0.03 *	Age Sex Baseline Cognitive status ApoE ε-4 status	MMKD alters gut microbiome composition, and SCFA levels correlate with improved AD biomarkers in CSF, suggesting potential dietary modulation of AD pathology.
Takeuchi and Kawashima, 2021 (Japan) [35]	Longitudinal cohort study **UK Biobank** European adults (no dementia diagnosis) **Total** (*n* = 340,000) **Dementia** **Total** (*n* = 900) **Follow-up** 5 years **Ethnicity** European	≥60	NR	NR	Self-reported dietary intake. Food categories: processed meat, poultry, beef, lamb, pork, oily fish, non-oily fish, fresh fruit, dried fruit, raw vegetables, cooked vegetables, cheese, cereal, tea, coffee, bread	HR for MD food associations and dementia incident. High bread intake, moderate meat and fish intake, low vegetable and fruit intake associated with a significant decrease in dementia onset risk. Poultry and cereal intake not significantly associated with dementia risk.	0.008 * 0.003 * 0.043 * 0.008 * 0.003 * 0.048 * NR	Sex Age Socioeconomic Education Income Physical activity BMI Height Alcohol consumption Smoking Race Depression Systolic blood pressure Diabetes, heart attack, cancer diagnosis	Findings inconsistent with the idea that the MD, which emphasizes high vegetable and fruit intake and low meat intake, is associated with a lower risk of dementia.
Shannon et al., 2021 (UK) [12]	RCT Older adults at risk of dementia **Total** (*n* = 108) **Ethnicity** British	55–74	Blood, urine, fecal	MoCA COWAT CFT Digit symbol substitution TMT A and B RAVLT Hayling sentence Completion Digit Span Supermarket Trolley Task Sea Hero Quest	MD	Feasibility and acceptability of a multidomain intervention, increase in MD score, increase in PA levels, assessment of various cognitive, neurological, vascular, and biological outcomes. **Primary outcome:** Adherence to the intervention.	NR	Age Sex Socioeconomic Education Baseline cognitive function Physical activity	Feasibility and acceptability of a multidomain intervention focused on MD and PA for dementia risk reduction in a UK cohort.
Shannon, 2023 (UK) [11]	Prospective cohort study Participants from UK Biobank **Total** (*n* = 60,298) **Ethnicity** British, Irish, or Caucasian	≥60	Blood	Dementia diagnosis using ICD-9 and ICD-10 codes from hospital records and death registries	MEDAS and PYRAMID scores	Higher MD adherence associated with lower dementia risk, independent of genetic predisposition. No significant interaction between MD adherence and PRS for dementia.	<0.001 * NS	Age Sex Socioeconomic Education Smoking Sleep duration Physical activity Energy intake	Higher MD adherence reduces dementia risk independent of genetic predisposition. MEDAS is a sensitive predictor of dementia risk.
Mamalaki et al., 2021 (Greece) [111]	Cross-sectional study HELIAD study Community-dwelling older adults ≥65 years **Total** (*n* = 1726) **Ethnicity** Greek	**Mean age:** 72.8 ± 5.8 years **Age range:** ≥65 years	NR	Neurological and neuropsychological assessment	69 item FFQ, MD adherence using the MedDiet score and total lifestyle indices	MD and social patterns positively associated with major cognitive domains and global cognitive functioning.	<0.001	Age Sex Education	MD lifestyle, incorporating diet, sleep, physical activity, and social interactions, is positively associated with cognitive function in older adults without dementia.

2-AG: 2-Arachidonoyl Glycerol, AD: Alzheimer’s Disease, ADAS-Cog: Alzheimer’s Disease Assessment Scale-Cognitive, ADNI-2: Alzheimer’s Disease Neuroimaging Initiative, AEA: Anandamide, AF: Animal Fluency, AHAD: American Heart Association Diet, AHEI: Alternate Healthy Eating Index, aMED: Alternate Mediterranean Diet, ANU-ADRI: ANU-Alzheimer’s Disease Risk Index, BCSB: Brief Cognitive Screening Battery, B-IADL: Brody Instrumental Activities of Living Scale, BMI: Body Mass Index, B-SEVLT: Brief Spanish English Verbal Learning Test, CANTAB: Cambridge Neuropsychological Test Automated Battery, CANTAB-PAL: Paired Associates Learning Test from the Cambridge Neuropsychological Test Battery, CDR: Clinical Dementia Rating, CDT: Clock Drawing Test, CERAD: Consortium to Establish a Registry for Alzheimer’s Disease, CFT: Cognitive Function Test, CHAP: Cognitive tests from Chicago Health and Aging Project, CI: Cognitively Impaired, CI: Confidence Interval, COWA: Controlled Oral Word Association Test, CSF: Cerebrospinal Fluid, CSI-D: Community Screening Instrument for Dementia, CVD: Cardiovascular Disease, CVLT-II: California Verbal Learning Test, DASH: Dietary Approaches to Stop Hypertension, DGLEA: Dihomo-Gamma-Linolenic Acid, DHA: Docosahexaenoic Acid, DHEA: Dehydroepiandrosterone, D-KEFS-VF: Delis–Kaplan Executive Function System-Verbal Fluency, DSST: Digit Symbol Substitution Test, DSM-IV: Diagnostic and Statistical Manual of Mental Disorders, eCBS: Endocannabinoids, EPA: Eicosapentaenoic Acid, EOD: Early-Onset Dementia, EPIC: European Prospective Investigation into Cancer and Nutrition, EVOO: Extra Virgin Olive Oil, FDG: Fludeoxyglucose, FCSRT: Free and Cued Selective Reminding Test, FFQ: Food Frequency Questionnaire, GDS: Geriatric Depression Scale, FIT: Fluid Intelligence Test, GMS: Geriatric Mental Schedule, HDL: High Lipid Lipoprotein, HEI: Healthy Eating Index, HVLT: Hopkins Verbal Learning Test, IST: Intelligence Structure Test, LDL: Low-Density Lipoprotein, LEA: Linoleoyl Ethanolamide, 3MS: Modified Mini-Mental State Examination, 7-MS: 7-minute Screen Test, MAP: Memory and Aging Project, MCI: Mild Cognitive Impairment, MCLHB-DRR: Motivation to Change Lifestyle and Health Behaviors for Dementia Risk Reduction, MD: Mediterranean Diet, MDP: Mediterranean Diet Pattern, MDS: Mediterranean Diet Score, MeDi Score: Mediterranean Diet Scoring Tool, MEDAS: Mediterranean Diet Adherence Screener, MIND: Mediterranean-DASH Intervention for Neurodegenerative Delay, MMKD: Modified Mediterranean Ketogenic Diet, MMSE: Mini-Mental State Examination, MNA: Mini Nutritional Assessment, MoCA: Montreal Cognitive Assessment, MUFA: Monosaturated Fatty Acids, MRI: Magnetic Resonance Imaging, MUFA: Monounsaturated Fatty Acid, NC: Normal Cognition, NINCDS-ADRDA: National Institute of Neurological and Communicative Disorders and Stroke and the Alzheimer’s Disease and Related Disorders Association, NPI: Neuropsychiatric Inventory, PCC: Posterior Cingulate Cortex, OEA: Oleoylethanolamide, OR: Odds Ratio, PA: Physical Activity, PD: Probable Dementia, PEA: Palmitoylethanolamide, PET: Positron Emission Tomography, PIR: Ratio of Family Income to Poverty, POEA: Palmitoleoyl Ethanolamide, PREDIMED: Prevention with Mediterranean Diet, PRS: Polygenic Risk Score, PUFA: Polyunsaturated Fatty Acids, RAVLT: Rey Auditory Verbal Learning Test, RCT: Randomized Controlled, rMED: Relative Mediterranean Diet, ROCF: Rey-Osterrieth Complex Figure, Trial, SCD: Subjective Cognitive Decline, SCF: Subjective Cognitive Function, SDMT: Symbol Digit Modalities Test, SEA: *N*-stearoylethanolamine, SFA: Saturated Fatty Acids, SFFQ: Semi-Food Frequency Questionnaire, SF-EMSE: Short-Form Extended Mental State Exam, STICS-m: Spanish Telephone Interview for Cognitive Status, TLI: Total Lifestyle Index, TMT: Trail Making Test, VPA: Verbal Paired Associates, WD: Western Diet, WHICAP: Washington Heights Inwood Columbia Aging Project, WHIMS: Women’s Health Initiative Memory Study, WMV: White Matter Volume, WDP: Western Diet Pattern, y: Years, NS: Non-significant, NR: Not Reported. Significant *p*-values are indicated in **bold** *.

**Table 3:** Characteristics of studies investigating the association of the Nordic Diet, dietary patterns, and intake in Alzheimer’s Disease, dementia, and at-risk individuals.

First Author, Year (Country)	Study Type, Subjects, and Ethnicity, N	Mean Age at Blood Collection ± SD Age Range (Years)	Sample Type	Cognitive Function Assessment	Exposure (Dietary Consumption, Dietary Patterns)	Clinical Outcome, Analysis, and Effect Estimation	*p*-Value	Cofounders	Clinical Conclusions
Shakersain et al., 2018 (Sweden) [30]	Population-based longitudinal cohort study Swedish National Study on Aging and Care in Kungsholmen (SNAC-K) **Younger cohort** (Aged: 60, 66, 72 years) **Older cohort** (Aged: 78, 81, 84, 87, 90, 93, 96, >99 years) **Men** (*n* = 871) (39.2%) **Women** (*n* = 1352) (60.8%) **Total** (*n* = 2223) **Ethnicity** Swedish	**Men** 69.5 ± 8.6 **Women** 71.3 ± 9.1	Blood	MMSE	98-item SFFQ questionnaire for NPDP adherence. **Low NPDP** (*n* = 720) **Moderate NPDP** (*n* = 779) **High NPDP** (*n* = 724)	Moderate-to-high adherence to NPDP associated with reduced decline in MMSE score. (β: 0.19, 95% CI: 0.14–0.24). The association was stronger with moderate-to-intense physical (β: 0.34, 95% CI: 0.2–0.45), mental (β: 0.29, 95% CI: 0.21–0.37), or social activities (β: 0.27, 95% CI: 0.19–0.34).	<0.001 * <0.001 * <0.001 * <0.001 *	Age Sex Education Marital status Total caloric intake Smoking BMI APOE ε4 status	An active lifestyle significantly enhances the protective effect of a healthy diet on cognitive function, reducing the risk of cognitive decline.
Wu et al., 2021 (Sweden) [31]	Prospective cohort study Dementia- and disability-free adults aged ≥60, **Female** (60.8%) **Male** (39.2%) **Total** (*n* = 2290) **Follow-up** 12 y **After follow-up** **Non-dementia** (*n* = 1074) **Died** (*n* = 518) **Disabled** (*n* = 614) **Dementia** (*n* = 84) **Ethnicity** Swedish	**Baseline** 70.8 ± 9.1 **Follow-up** **Died** 83.5 **Disabled** 77.8 **Dementia** 86.5	Blood	Standard criteria for dementia, Katz’s ADL for disability	98 item FFQ NPDP **Low NPDP** (*n* = 746) **Moderate NPDP** (*n* = 757) **High NPDP** (*n* = 787)	**Low/moderate/high NPDP** Age Female Education BMI Smoking Alcohol consumption Social network Mental leisure activity Social leisure activity Physical activity.	<0.01 * <0.01 * <0.01 * <0.01 * <0.01 * <0.01 * <0.01 * <0.01 * <0.01 * <0.01 *	Age Sex Education BMI Smoking Alcohol consumption Physical activity Chronic diseases	High NPDP adherence is associated with 3.80 years longer life without dementia and disability. NPDP adherence associated with a 20% higher probability of dementia- and disability-free survival, prolonged lifespan without mental and physical disability by 1.24 years, a favorable lifestyle profile.
Shakersain et al., 2018 (Sweden) [29]	Prospective cohort study Swedish National Study on Aging and Care in Kungsholmen (SNAC-K) Dementia-free adults **Men** (*n* = 871) (39.2%) **Women** (*n* = 1352) (60.8%) **Total** (*n* = 2223) **Follow-up** 6 y **Ethnicity** Swedish	**Men** 69.5 ± 8.6 **Women** 71.3 ± 9.1	Blood	MMSE at baseline and follow-ups	98-item SFFQ NPDP, MD-DASH Intervention for MIND, Score DASH, and Baltic Sea Diet	Moderate (β = 0.139, 95% CI: 0.077–0.201) to high (β = 0.238, 95% CI 0.175–0.300). NPDP adherence associated with less cognitive decline. High NPDP adherence associated with lower risk of MMSE decline to ≤24 (HR = 0.176, 95% CI 0.080–0.386).	<0.001 * <0.001 * 0.002 * <0.001 * 0.049 *	Age Sex Education Marital status Smoking Physical activity BMI APOE ε4 status	NPDP a better Individuals with high adherence to all dietary patterns had the highest level of education, were physically very active, and a lower proportion were current smokers.

BMI: Body Mass Index, CI: Confidence Interval, DASH: Dietary Approaches to Stop Hypertension, FFQ: Food Frequency Questionnaire, MIND: Mediterranean-DASH Intervention for Neurodegenerative Delay, MMSE: Mini-Mental State Examination, NPDP: Nordic Prudent Diet Pattern, SFFQ: Semi-Food Frequency Questionnaire, SNAC-K: Swedish National Study on Aging and Care in Kungsholmen, y: Years, NS: Non-significant, NR: Not Reported. Significant *p*-values are indicated in **bold** *.

## Supplementary Materials

The following supporting information can be downloaded at www.mdpi.com/article/10.3390/nu17020336/s1, Table S1: PRISMA checklist for systematic reviews; Table S2: Overview of included studies; Table S3: Quality assessment of risk bias for controlled intervention studies; Table S4: Quality assessment of risk bias for case–control studies; Table S5: Quality assessment of risk bias for observational cohort, longitudinal and cross-sectional studies; Supplementary Data S1: Summarized study results. Reference [134] is cited in the supplementary materials.
