# Supplementary material for: Dietary Intake, Mediterranean and Nordic Diet Adherence in Alzheimer’s Disease and Dementia: A Systematic Review
Source: Nutrients. 2025 Jan 17;17(2):336. doi: 10.3390/nu17020336 (PMC11767999; doi:10.3390/nu17020336)
Supplement: Supplementary file 1 [file nutrients-17-00336-s001.zip › nutrients-3407109-supplementary/Supplementary Data S2.pdf]

3. Interventions

3.1 Micronutrients and macronutrients

Micronutrients and Macronutrients including vitamins such as B6, B12, C, D, and E, along with minerals like zinc, iron, and magnesium, play crucial roles in various biochemical processes in the brain and have vital roles in maintaining neuronal health, cognitive health and preventing neurodegenerative diseases. For instance, the B vitamin class are essential for maintaining brain health by reducing homocysteine levels, which, when elevated, and are associated with cognitive decline and AD. Macronutrients, such as carbohydrates, proteins, and fats, are fundamental for brain function. Omega-3 fatty acids, particularly DHA and EPA found in fish oil, are critical for maintaining neuronal membrane integrity and function. Protein sources, including lean meats, fish, and legumes, provide amino acids necessary for neurotransmitter production. Carbohydrates, particularly those with a low glycemic index, provide a steady supply of glucose, the brain's primary energy source, ensuring sustained cognitive function.

Zhao et al. (2020) [49] conducted a longitudinal study with healthy older adults and found that higher vitamin D intake from food sources is associated with a decreased risk of dementia, indicating the importance of vitamin D in cognitive health maintenance and disease prevention. Study participants within the highest tertile of vitamin D intake from various food sources such as salmon, sardines, dairy and plants milks fortified with vitamin D had a decreased risk for dementia in comparison to individuals in the lowest tertile.

Kuhn et al. (2021) [39] conducted a 12-week RCT trial to investigate fish intake and cognition. The intervention group received canned pilchards and fish spread while the control group received canned meatballs and soy every week. Individuals in the intervention group had increased CASI scores in comparison to control group. Moreover, the intervention group had increased omega-3, RBC-EPA and DPA concentrations compared to the control group. Twelve weeks of additional fish intake improved cognition in resource-limited elderly people.

3.2 Physical Activity, Dietary Adherence and Cognition

A study by Ahn et al., 2022 [60] investigated if a combination of high-intensity physical activity and adherence to the MIND diet is possibly correlated with improved cognition in comparison to behavior alone or no behavior respectively. The study identified that PA-/MIND+ was associate with better global cognition and reduced odds of cognitive decline vs PA-/MIND-. The PA +/MIND + associated with better global cognition ($d = 0.98$, 95% CI: 0.59–1.36) and lower odds of cognitive decline ($OR = 0.69$, 95% CI: 0.50–0.94) compared to PA −/MIND. Therefore, combining high-intensity physical activity and MIND is associated with better cognitive health. Multi-domain lifestyle interventions combining diet, physical activity, and other lifestyle factors can offer comprehensive benefits for cognitive health were investigated by McMaster et al. 2018 [97] where a randomized controlled trial was conducted in MCI or SCD individuals. The study demonstrated that high intervention adherence (diet, cognitive engagement, and physical activity) significantly reduced cognitive decline in individuals with MCI and SCD.

3.3 Dietary Intake and Patterns and Cognition

Mediterranean Diet

The role of the MD, dietary intake and patterns is well investigated in AD and dementia patients compared to other neurodegenerative diseases through numerous studies as identified within this systematic review. Various studies have revealed MD adherence to be associated with decreased risk of cognitive impairment or slower cognitive decline in individuals at risk of AD and dementia. Moreover, MD adherence has also been shown to reduce AD risk and improve cognitive function due to the high concentration levels of antioxidants, anti-inflammatory compounds, and healthy fats that contribute to the neuroprotective effects observed in neuronal cells.

A study by Chan et al. 2015 [36] identified that higher MD adherence was associated with a reduced risk of cognitive impairment in older Chinese women due to the higher intake of vegetables and fruits. Interesting, the same study identified that snacks, drinks and milk products were associate with reduced risk of cognitive impairment. However, no association was found between MDS and dietary patterns identified and cognitive function in Chinese men.

A longitudinal study conducted by Charisis et al. 2021 [37] conducted in non-demented Greek individuals, in which 62 dementia cases were identified after follow-up identified that study participants, who had higher MD adherence has a 72% lower risk of dementia and slower cognitive decline. Therefore, high MD adherence was associated with a decrease in dementia risk and cognitive decline in a population following a traditional MD.

Extra virgin olive oil (EVOO) is a nutritious component with high phenolic content, such as Oleic acid, it is high in MUFAs and low in SAFs, which adds to its antioxidant, anti-inflammatory, neuroprotective properties. A study by, Valls-Pedret et al., [38], investigated the role of EVOO and mixed nuts in cognitively healthy volunteers for a period of 6 years. The MD+EVOO group scored better on the RAVLT and Color Trail Test part 2 compared to controls. Additional cognitive tests such as MMSE, Digit Span, VPA were identified to be changed from baseline measurements in the MD+EVOO and MD+Nuts group compared to controls. Moreover, study participants in the in the MD+EVOO and MD+Nuts group demonstrated changes in frontal and global cognition and compared to control group. The above-mentioned study indicates improved cognitive function in individuals' supplements with EVOO or nuts.

A study by Zupo et al., [40] investigate a plant-based diet consisting of coffee, vegetable and foods of vitamin A sources. The study found that plant-based foods, coffee and vegetables were inversely associated with cognitive impairment, while alcohol consumption showed to be detrimental. Interestingly, the study identified red meat to beneficial although it is usually considered to be harmful on cognitive function. The study confirmed that a MD pattern based on high consumption of fruits and vegetables with low alcohol consumption my prevent or delay cognitive impairment/ However, more research is required to determine the effect of red meat consumption on cognitive function.

Martínez-Lapiscina et al., [41] investigated the effect on cognition with a controlled MD nutritional intervention, this included MD+EVOO, MD+Nuts and MD+Low Fat diets. Improved post-trial cognitive performance was observed in all cognitive domains, specifically regarding fluency and memory tasks were observed for all study participants in the MD+EVOO group vs controls. Moreover, once adjusting for cofounders the MD+EVOO demonstrated a reduced MCI vs control group. While the MD+Nuts group did not differ from that of the MD+Low Fat control group. The study highlighted that higher MD adherence with EVOO was associated with better visual and verbal memory performance and overall better cognitive function and lower MCI in comparison to control diet, thus emphasizing the diet's protective effects against cognitive decline.

A previous study by, Muñoz-García et al., [54] investigate the Mediterranean Diet Pattern (MDP) and Western Diet Pattern (MDP) using a FFQ and cognition was performed using the Spanish version of the modified Telephone Interview of Cognitive Status (STICss-m). The WDP was associate with a negative STICS-m scores, while MDP was associated with a positive STICS-m scores. The following, indicates that higher MD adherence was associated with a decrease in cognitive function therefore, lowering the incidence of dementia a six-year period. While the WDP was associated with a greater increase in cognitive function, indicating its role as potentially increasing dementia incidence.

A study by Scarmeas et al., [69] found that higher adherence to the MD is associated with a lower risk of AD, independent of vascular comorbidities. The results showed that higher MD adherence was significantly associated with a lower risk of AD. Specifically, participants in the highest MD adherence tertile had 68% lower odds of developing AD compared to those in the lowest tertile. The association remained robust even after adjusting for various potential confounders and when vascular risk factors were added to the models. These findings suggest that the protective effect of the MD on AD risk is not mediated by vascular comorbidity, implying the involvement of other mechanisms, such as oxidative stress or inflammation.

Another study by Scarmeas et al., [76], individuals with AD were followed for an average of 4.4 years to assess the impact of adherence to the MD on mortality. The results indicated that higher adherence to the MD was associated with a lower risk of death. Specifically, each additional point on the MD adherence scale was linked to a 21% reduction in mortality risk This association remained significant after adjusting for potential confounders, with the highest level of MD adherence corresponding to a survival benefit of approximately 3.91 additional years compared to those with the lowest adherence. The findings suggest a dose-response relationship, where better adherence to the MD is linked to improved survival in AD patients.

One study by Scarmeas et al., [64], conducted a prospective study that investigate MD adherence and physical activity in reducing AD risk. The study, found that higher MD adherence and some forms of physical activity were in fact associated with a lower AD risk, in comparison to individuals who either had lower or did not adhere to the MD and there was physical activity involved, had an 19% increased risk of AD. Therefore, both higher MD adherence and physical activity are independently correlated with a reduced risk for AD.

Scarmeas et al., [82] investigated the association between MD and MCI. Study participants in the moderate MD adherence tertile had a decrease chance of developing MCI compared to those in the low tertile. Individuals who had high MD adherence had less risk of developing MCI. Higher MD adherence was associated with a decreased risk (48%) of developing MCI and a reduced likelihood for MCI to AD conversion. While individuals with moderate MD adherence had a 45% less chance of developing AD. Therefore, high MD adherence was associate with a reduced likelihood of developing MCI and progression of MCI to AD, this suggests a protective effect by dietary interventions.

Martín et al. [51] conducted a comparative study aimed to investigate the association between food habits, body composition, lifestyle, and gustatory function in individuals with AD compared to healthy controls. The results revealed that AD patients had lower body mass index (BMI) and weight, and slept more hours, with significant differences observed between groups. AD patients also showed poorer adherence to exercise and the Mediterranean diet. Additionally, gustatory function was impaired in AD patients compared to controls. Overall, the findings suggest that individuals with AD experience worse outcomes in terms of anthropometric measurements, lifestyle habits (diet and exercise), and sensory function.

The study population-based cross-sectional study by Samuelsson et al., [52] investigated the relationship between dietary patterns and cerebrospinal fluid (CSF) biomarkers associated with AD in dementia-free older adults. Higher adherence to a WDP was significantly associated with increased odds of having total tau pathology and preclinical AD (Aβ42 and tau pathology). Furthermore, no

significant associations were found between the other dietary patterns (Mediterranean/prudent, high-protein/alcohol, high-fat) and CSF biomarkers, either in unadjusted or adjusted models.

A previous study by, Blumenthal et al. 2017[129] was interested to determine the relationship of lifestyle factors (DASH and MD adherence) and neurocognitive functions in older individuals with vascular risk and cognitive impairment without dementia (CIND). Lifestyle factors such as aerobic exercise and daily physical activity were associated with improved executive functioning, processing speed and verbal memory. DASH diet adherence was associated with improved verbal memory. Furthermore, Increased hsCRP and FSRP score was linked with poor executive function, processing speed and verbal memory. The study observed that the correlation between verbal memory and DASH diet was strongly influenced by low total dietary fat intake. Therefore, physical activity and adherence to the DASH diet and MD were associated with better neurocognitive performance, suggesting that a healthy lifestyle could reduce neurocognitive decline.

Gu et al. 2010 [73] investigated the MD, inflammatory and metabolic biomarkers and the risk of AD. The authors observed that better adherence was associate with decreased hsCRP concentration, but no association was observed with fasting insulin and adiponectin. Additionally, individuals in the highest tertile of MD adherence had a 34% decreased risk of developing AD. However, the association between better MD adherence and low AD risk was not mediated by hsCRP, fasting insulin and adiponectin. Other inflammatory and metabolic pathways not observed by these biomarkers, or other non-inflammatory and non-metabolic pathway by be relevant and contribute to the MD-AD association.

Another study by Gu et al. 2015 [130] investigate if higher MD adherence is related to larger brain volume and cortical thickness as measured by MRI. The study observed that high MD adherence (5-9) in comparison to lower MD adherence showed larger TBV, TGMV and TWMV. Moreover, high fish and low meat intake was associate with larger TGMV, while lower meat intake was associated with an increased TBV. High fish intake was correlated with a larger mCT. It is evident that the volumes of the cingulate cortex, parietal and temporal lobes and hippocampus and the CT of the superior frontal regions were correlated with dietary factors. Higher MD adherence was associated with larger TBV and lower brain atrophy, emphasizing the diet's role in maintaining brain structure.

The prospective, longitudinal study by Walters et al., [44] aimed to explore the associations between lifestyle and vascular risk factors and changes in AD biomarkers and global cognition in middle-aged, asymptomatic individuals at risk for AD. The study showed that adherence to a Mediterranean-style diet was associated with slower decline in glucose metabolism in the posterior cingulate cortex and, to a lesser extent, in the frontal cortex. However, diet had no effect on amyloid or cortical thickness changes. In contrast, higher baseline plasma homocysteine levels were linked to faster cognitive decline, independent of lifestyle and biomarker measures. No other lifestyle or vascular risk factors were significantly associated with changes in AD biomarkers or cognition.

MD, insulin sensitivity in terms of cognitive function was investigated by Mosconi et al., 2018 [3] in middle-aged individuals. The study found that MD and insulin sensitivity positively associated with MRI-based cortical thickness and, EC thickness, intellectual enrichment and overweight respectively, which negatively impacts cognitive performance resulting in cognitive dysfunction.

Chen et al., [46] examined the combined effects of particulate matter (PM2.5) exposure and adherence to a MIND diet on white matter volume (WMV) in the brains. The study uncovered that exposure to PM2.5 was associated with lower white matter volume, indicating brain aging, but this effect was stronger among women whose diets were less consistent with the MIND dietary pattern. Adherence to a MIND-like diet may offer protective benefits against the neurotoxic effects of PM2.5 exposure on brain health.

The prospective study by Morris et al., [26] investigated the relationship between adherence to three dietary patterns (MIND, DASH, and MD) and the risk of developing AD. High adherence to the MIND, DASH, and Mediterranean diets is associated with a reduced risk of developing Alzheimer’s disease,

with moderate adherence to the MIND diet also showing potential benefits in lowering AD risk. These findings suggest that dietary patterns emphasizing brain-healthy foods may play a role in AD prevention.

The cohort study by Franzon et al., [48] aimed to examine the longitudinal associations between aging with preserved functionality (independent aging) and lifestyle factors, dietary patterns, and cardiovascular risk factors in Swedish men. Lifestyle factors such as never smoking, high adherence to a Mediterranean-like diet, and maintaining a healthy weight were associated with both survival and independent aging at age 85 or older in men. These findings suggest that lifestyle choices made in middle age can significantly impact longevity and functional independence in later years.

The cross-sectional study Wesselman et al., [56], investigated the associations between dietary patterns and cognitive functioning in elderly individuals free of dementia. In fully adjusted models, adherence to both the Mediterranean and MIND diets was associated with better memory performance. The alcoholic beverages PCA component was positively associated with cognitive function in most domains. When participants with MCI were excluded (n = 60), the Mediterranean and MIND diets remained significantly associated with better language function. The associations with the alcoholic beverage's component were weakened, but most remained significant. The study found that adherence to the Mediterranean and MIND diets, as well as some data-driven dietary patterns, was linked to better memory and language function in elderly individuals.

This cohort study by Moustafa et al., [57],aimed to investigate the association between adherence to a MD and cognitive performance among Hispanic or Latino adults. The results showed that after adjusting for relevant covariates, individuals in the high adherence group performed better than those in the low adherence group on cognitive tests, including B-SEVLT Sum, B-SEVLT Recall and Global cognition. Moreover, the high adherence group showed less cognitive decline compared to the low adherence group in learning and memory. However, no significant differences were observed in word fluency, Digit Symbol Substitution Test (DSST) scores, or global cognition. Overall, the study concluded that high MD adherence was associated with better cognitive performance and less decline in learning and memory over a 7-year period among middle-aged and older Hispanic or Latino adults.

The longitudinal cohort study by Mamalaki et al.,[59], aimed to examine the association between a Total Lifestyle Index (TLI), which includes MD adherence, sleep duration, physical activity, and engagement in activities of daily living, with cognitive health and dementia risk in older adults. Participants who developed dementia had lower TLI scores compared to those with normal cognition. The study suggests that greater adherence to a healthy lifestyle pattern, as reflected by a higher TLI, is associated with slower cognitive decline and a reduced risk of dementia in older adults. These findings emphasize the importance of maintaining a healthy lifestyle, including diet, physical activity, and daily engagement, in promoting cognitive health in aging populations. Another study by Mamalaki et al., [112], was a cross-sectional study, with the aim to investigate the association of lifestyle patterns, reflecting Mediterranean lifestyle components, with cognitive performance in a cohort of older adults (≥65 years) without dementia. The study identified that higher adherence to the lifestyle factors of high MD adherence, physical activity, daily functioning and good sleep quality were positively associated with better cognitive performance in almost all major cognitive domains and overall global cognitive functioning.

Wengreen et al., [61] conduct a study to investigate the DASH and MD patterns regarding age-related cognitive alternations. High DASH and MD scores were associated with increased 3MS scores. Individuals in DASH quintile 5 averaged 0.97 points higher than those in quintile 3, while for MD adherence the quintile difference was 0.94. The high intake of whole grains, legumes and nuts were also associate with increased 3MS scores when comparing quintile 5 and quintile 1. This highlights the neuroprotective nature of these foods. Higher DASH and MD adherence to was associated with consistently higher cognitive function over an 11-year period.

The study by Talegawkar et al., [62] ,investigated the association between adherence to a Mediterranean-style diet and the risk of frailty in older adults. The study suggests that higher adherence to a Mediterranean-style diet is inversely associated with the development of frailty in community-dwelling older adults. The diet appears to specifically reduce the risk of low physical activity and low walking speed, both of which are components of frailty. These findings highlight the potential benefits of a Mediterranean-style diet in promoting healthier aging and preventing frailty in older populations.

The aim of the prospective cohort study by Tangey [63], aimed to examine the relationship between adherence to the DASH and MD and the rate of cognitive decline. The study showed that adherence to the DASH and MD were found to be associated with slower rates of cognitive decline in older adults. These results support the hypothesis that these dietary patterns may help preserve cognitive function in aging populations.

Calil et al., [65], conducted a cross-sectional study with the aim to investigate the association between MD and MIND adherence and cognitive performance in elderly individuals with different cognitive profiles. The study found no significant differences in body mass index (BMI) or adherence levels between the clinical groups (NC, MCI, AD). MIND and MD adherence was positively associated with higher cognitive performance scores on the MMSE and BCSB learning tasks, but this association was found only in the NC group. The results suggest that dietary patterns may benefit cognitive function in seniors, particularly in low- to middle-income countries. However, further studies are needed to confirm whether these diets could have similar benefits for individuals with cognitive impairments such as MCI or AD.

The prospective cohort study by Féart et al., [66], aimed to analyze the relationship between plasma fatty acids and adherence to the Mediterranean diet in a cohort of elderly French adults. Positive assocations were observed for plasma DHA, EPA + DHA index, and total n-3 PUFA levels were positively associated with MD adherence. Moreover, the association between plasma EPA and MD adherence was stronger in individuals who were non-carriers of the APOE-ε4 allele. However, o associations were found between MD adherence and saturated fatty acids (SFA) or total monounsaturated fatty acids (MUFA). The results suggest that the protective effects of the Mediterranean diet on cognitive function may be partly mediated by higher plasma DHA levels and a lower n-6:n-3 PUFA ratio. The findings also indicate that the relationship between MD adherence and plasma EPA levels may depend on APOE-ε4 status, with non-carriers showing stronger associations.

Larsson and Wolk, [67] conducted a prospective cohort study with the aim to investigate whether lifestyle factors and sleep duration are associated with the risk of late-onset dementia in older adults. No significant associations were found between an overall healthy diet, alcohol or coffee consumption, physical activity, and dementia incidence. No clear evidence that major lifestyle factors (aside from smoking) or sleep duration influence the risk of dementia. The observed association between extended sleep duration and increased dementia risk appears to be due to reverse causation, as it did not persist after excluding early diagnosed cases. Smoking, however, was associated with a modest increase in dementia risk.

The prospective cohort by Margara-Escudero et al.,[33], aimed to examine the association between egg consumption and the risk of dementia in a Mediterranean population. No significant association was found between egg consumption and the risk of total dementia or AD in the entire cohort. This study suggests that egg consumption may be associated with a reduced risk of dementia, particularly AD, in individuals with low MD adherence. In contrast, egg consumption did not appear to have an impact on dementia risk in individuals with moderate or high adherence to the MD. These findings highlight the potential role of dietary patterns in modulating the neuroprotective effects of specific foods like eggs.

Gardener et al., [70], conducted a cross-sectional study with the aim to investigate the relationship between adherence to the MD and the risk of AD and MCI in a large cohort of elderly Australians. Overall, AD participants had significantly lower MD adherence compared to healthy controls, while MCI participants also exhibited lower adherence compared to healthy controls. In healthy controls, higher adherence to the Mediterranean diet was associated with a significant change in MMSE over 18 months. These findings suggest that higher adherence to the Mediterranean diet may be linked to a lower risk of cognitive decline and could potentially slow cognitive deterioration in healthy elderly individuals.

Rocaspana-García et al., [72], conducted a cross-sectional study to investigate the nutritional status of AD patients at different stages of the disease, and to explore the correlation with cognitive, functional, and behavioral variables, and evaluate their adherence to the MD. The study found, poor nutritional status **is** common in AD patients, with high rates of malnutrition and low MD adherence. Nutritional status was correlated with cognitive function, functional status, and behavioral symptoms, as well as caregiver burden. The findings suggest that improving nutritional habits, particularly increasing MD adherence, could potentially benefit both cognitive outcomes and caregiver burden in Alzheimer's disease patients.

Shannon et al. 2019 [87] aimed to examine the associations between the MD and cognitive function in the older UK population to further investigate if association differ between individuals with high to low CVD risk. Higher MD adherence to was associated with better global cognition, verbal episodic memory, and processing speed. Moreover, a reduced risk of poor verbal episodic memory, processing speed and prospective memory were also observed in individuals with a higher MD adherence compared to the low MD adherence individuals. This emphasizes the diets comprehensive benefits in terms of cognitive performance. An additional study by Shannon et al., 2023 [12], investigate MD adherence and dementia risk. Overall, the study found that higher MD adherence was associated with a lower risk of dementia, independent of genetic risk factors, highlighting the potential of dietary interventions as part of dementia prevention strategies. This study underscores the importance of diet in reducing dementia risk, regardless of an individual's genetic predisposition.

The longitudinal cohort study by Takeuchi and Kawashima, [35], aimed to investigate the relationship between adherence to a Mediterranean-style diet and the risk of developing frailty in a community-dwelling population of older adults. After a 6-year follow-up, participants with higher MD adherence (score ≥ 6) had significantly lower odds of developing frailty compared to those with lower adherence (score ≤ 3). In community-dwelling older adults, higher adherence to a Mediterranean-style diet was inversely associated with the development of frailty. Specifically, higher adherence to the diet was linked to a reduced risk of low physical activity and slower walking speed, key components of frailty. These findings suggest that promoting MD adherence may help prevent frailty and improve functional health in older adults.

Ye et al., 2013 [80], conducted a cross-sectional study with the study aimed to examine the associations between dietary quality, as assessed by both MD and HEI-2005, and cognitive performance in middle-aged Puerto Rican adults. In middle-aged and older Puerto Rican adults, higher adherence to either the MD or the USDA dietary guidelines is associated with improved cognitive performance and a lower likelihood of cognitive impairment. These findings suggest that both dietary patterns may offer protective benefits for cognitive health, irrespective of ethnic background, supporting the importance of diet in cognitive aging.

A longitudinal cohort study by Roberts et al., [81], aimed to investigate and explore the associations between the components of MD and the MD score with the risk of MCI. High MD adherence score was associated with a reduced risk of incident MCI or dementia. Furthermore, the study suggests that components of the MD, including vegetable intake and a high ratio of unsaturated fats to saturated fats, may be beneficial for cognitive function and may help reduce the risk of mild cognitive impairment.

While high adherence to the MD showed a trend towards reduced cognitive decline, further studies with larger sample sizes may be needed to confirm these findings.

Trichopoulou et al., [83] conducted a prospective cohort study, this study aimed to assess whether MD adherence is inversely associated with cognitive decline among the elderly population in Greece, and whether any MD component plays a key role in this association. The study observed that participants with high MD adherence were significantly less likely to experience mild cognitive decline compared to those with low adherence. Furthermore, the study found that high MD adherence was also associated with a significantly reduced risk of substantial cognitive decline. Moreover, higher vegetable consumption appears to play a key role in this protective effect, potentially working in synergy with other components of the MD.

A cohort study was conducted by Dobreva et al., [84], the following study investigates the associations between various MD components and all-cause dementia risk in the UK Biobank cohort. Moderate fish consumption (2.0–3.9 times per week) was associated with a significantly lower risk of dementia compared to that of no fish consumption. Consumption of 1.0–1.9 servings of fruit per day was associated with a reduced dementia risk compared to no fruit consumption. Lastly, no other MD components (vegetables, processed meat, unprocessed red meat, unprocessed poultry, cheese, whole grains) showed a significant association with dementia risk after adjusting for other dietary factors. The findings suggest that fish consumption may be the key dietary factor driving the beneficial effects observed with adherence to the MD.

Soldevila-Domenech et al., [85], conducted a RCT focusing on APOE-ε4 carriers, they found that MD intervention led to significant improvements in memory and global cognition, particularly in men compared to women. This study highlights the importance of considering sex differences in dietary interventions, as genetic factors may interact differently with dietary components depending on gender. Furthermore, elevated plasma levels of 2-AG and AEA were positively associated with improved memory and executive function in both sexes, with stronger associations observed in women and APOE-ε4 carriers**.** In contrast, OEA and PEA concentrations were less consistently associated with cognitive changes, though higher levels of OEA were linked to improvements in processing speed**.**

Nagpal et al. [111] conducted an RCT showing that a MMKD altered gut microbiome composition and was associated with improved AD biomarkers in CSF, particularly in individuals with genetic risk factors for AD. This study suggests that dietary interventions can influence gut-brain axis interactions, potentially modifying disease risk in genetically predisposed individuals. The MMKD seems to alter the gut microbiome composition and SCFA levels correlate with improved AD biomarkers in CSF, suggesting potential dietary modulation of AD pathology.

The population-based cohort study by Anastasiou et al., [88], aimed to explore the association between MD adherence and cognitive function in the elderly Greek population which is part of the *Hellenic Longitudinal Investigation of Ageing and Diet* (HELIAD) study. Higher MD adherence was significantly associated with better cognitive function, mainly in areas of memory and executive function. The study found a clear dose-response relationship were a higher MD score, resulted in a better cognitive performance. Furthermore, a higher consumption of fruits, vegetables, fish, and olive oil, were positively correlated with improved cognitive outcomes. Conversely, higher intake of processed foods and red meats was associated with worse cognitive performance.

A population-based cohort study was conducted by Nicoli et al., [90] with the aim to investigate the association between adherence to the MD and both prevalent and incident dementia in the older

population. The cross-sectional findings, found greater MD adherence and a higher consumption of foods such as eggs, fruits and vegetables and carbohydrates were associated with a lower dementia prevalence. While the longitudinal findings, observed a higher intake of legumes was correlated with a lower incidence of dementia, the hazard ratios (HR) for dementia incidence in the higher vs lower tertiles were as follows Legumes: Hazard ratio = 0.68 (95% CI: 0.47–0.97) and portions per week: Hazard ratio = 0.66 (95% CI: 0.46–0.95). The results of the study, suggest that higher MD adherence and legume consumption, lowers the risk of developing dementia.

The case-control study by Filippini et al., [91], aimed to investigate the relationship between dietary habits and the risk of developing early-onset dementia by examining dietary patterns and specific food intake, the study aimed to identify which foods or dietary patterns may influence the risk of EOD (early onset dementia). In terms of dietary intake, the study found that a high intake of dairy products (greater than 400g/day) was associated with an increased risk of EOD, furthermore, a higher EOD risk was observed in individuals consuming more than 350 g/day of cereals. Regarding fish and seafood, the study found no significant association with EOD risk. However, a U-shaped relationship was observed with canned fish, that suggests a potential increased risk with high consumption. An inverse relationship was found with fresh fish, where greater consumption was related with a reduced EOD risk. The study found a strong inverse relationship between the intake of vegetables (especially leafy greens) and EOD risk when consumption exceeded 100 g/day whereas citrus fruits and dry fruits also showed an inverse association with EOD risk. Generally, sweet consumption was not associated with EOD risk. However, dry cake and ice cream were positively related to EOD risk while chocolate products were inversely related to EOD risk. Regarding, beverages, no significant associations were found between beverage consumption and EOD risk, apart from coffee, moderate coffee intake was associated with a lower risk, whereas very high or low consumption showed no benefit. Regarding dietary adherence, the study found that MIND diet adherence was inversely related to EOD risk. The Mediterranean and DASH diets displayed an inverse relationship with EOD risk, but this was evident at very high levels of adherence.

Another population-based cohort study, conducted by Crom et al., [92], aimed to examine the relationship between MIND diet adherence and dementia risk across cumulative time periods, this allowed researchers to again insight into how dietary habits over time may contribute to dementia risk. At follow-up from baseline I (mean 15.6 years), during this period, 1188 participants developed dementia. Furthermore, a higher MIND diet score at baseline I was associated with a lower risk of dementia over the first 7 years of follow-up (hazard ratio [HR] for 1 SD increase in MIND diet score = 0.85, 95% confidence interval [CI] = 0.74–0.98). However, the association weakened and disappeared over longer follow-up periods. While a follow-up from Baseline II (mean 5.9 years), identified 248 participants developed dementia. The study found that a higher MIND diet score at baseline II was strongly associated with a lower risk of dementia over every follow-up interval. The association remained significant but slightly attenuated over time. Specifically, for a 7-year follow-up, the HR for each 1 SD increase in MIND diet score was 0.76 (95% CI = 0.66–0.87). This study supports the idea that better adherence to the MIND diet is associated with a decreased risk of dementia, especially in the first few years after dietary assessment

A cross-sectional study was conducted by Vassilaki et al., [93], with the aim to provide insights whether MD adherence influences amyloid-β accumulation in the brain. Higher MD adherence was significantly associated with lower amyloid-β deposition. In addition, the study identified that certain dietary components presented associations with Aβ deposition, this includes, higher vegetable consumption to be associated with lower amyloid-β deposition (beta = -0.043), greater intake of vitamin A was also associated with lower amyloid-β levels (beta = -0.041), beta-carotene was linked to reduced amyloid-β deposition (beta = -0.039) and moderate alcohol intake, participants with moderate alcohol intake showed lower amyloid-β deposition (beta = -0.074). The study suggests that dietary patterns, particularly the MD, may play a protective role against early biomarkers of AD.

The prospective cohort study, by Bhushan et al., [96], aimed to assess the long-term association between MD adherence and self-reported subjective cognitive function (SCF) in a large cohort of middle-aged males. Regarding MD adherence, 38% participants had moderate SCF scores, and while 7.3% had poor SCF scores based on their previous baseline assessments. In terms of an association between MD and SCF, in comparison to males in the lowest MD score quintile, those in the highest quintile had 36% lower odds of reporting a poor SCF score (odds ratio [OR] = 0.64, 95% CI 0.55–0.75) and 24% lower odds of reporting a moderate SCF score (OR = 0.76, 95% CI 0.70–0.83). The study found that long-term MD adherence was strongly associated with better SCF.

Puente-González et al., [99] conducted an RCT, the primary aim of the study was to evaluate the short- and medium-term effects (over 6 months) of a multicomponent physical exercise program combined with a MD on bone mineral density (BMD), fall risk, balance, and gait in AD patients. The intervention group in comparison to the controls, showed improved BMD and this was observed in the calcaneal bone. Moreover, improved gait and balance performance was also observed due to the combined physical exercise program, a decreased fall risk was also seen in the intervention group due to the improved physical function and bone health.

The study by Ntanasi et al., [100], aimed to investigate the association between MD adherence and frailty in the older adult population. The found that higher MD adherence was associated with lower odds of frailty. This suggests that dietary patterns such as the MD, may play an important role in preventing or delaying frailty in aging populations.

The study performed by Enrique de la Rubia Ortí, [102], aimed to assess the effects of a coconut oil-enriched MD on cognitive functions in AD patients and investigate whether there are differences in outcomes based on disease stage or sex. The study found that a coconut oil-enriched MD showed positive effects on cognitive function, predominantly in episodic and semantic memory and temporal orientation. Positive effects were more evident in females with mild-moderate stages of AD, some improvement was also observed in males and patients in more severe stages of the disease. The study suggests that a coconut oil enriched MD may improve cognitive functions in patients with Alzheimer's disease. These improvements appear to be influenced by both sex and disease severity, with mild-moderate females showing the most notable cognitive benefits. However, further studies are needed to confirm these findings and explore the potential of coconut oil as a non-pharmacological adjunct in Alzheimer's care.

The cross-sectional study by Ballarini et al., [106], aimed to investigate whether MD adherence is associated with cognitive functions and *in vivo* biomarkers related to AD. Specifically, the relationships between MD adherence and brain volume, cognitive performance, and AD biomarkers, such as amyloid and tau pathology. The study identified that higher MD adherence was associated with larger mediotemporal gray matter volume. Furthermore, improved memory performance was correlated to higher MD adherence. In terms of AD biomarkers, higher MD adherence was associated with lower levels of amyloid (Aβ42/40 ratio, $\beta \pm SE = 0.003 \pm 0.001$) and lower phosphorylated tau (pTau181) pathology ($\beta \pm SE = -1.96 \pm 0.68$). The findings highlight the potential of dietary interventions in protecting against AD and preserving cognitive health.

Encarnación Andreu-Reinón [109], conducted a prospective cohort study, the aim of the study was to evaluate the protective role of MD adherence and dementia incidence in large cohort over a long follow-up. The study found that the overall dementia risk in individuals with high MD adherence in comparison to low MD adherence was correlated with a 20% lower dementia risk. In terms of sex, women showed a promising association between high rMED adherence and a reduced risk of non-AD, whereas in men, no statistically significant association was observed between the MD and AD risk. In

addition, researchers also investigated the effect of educational level, an association between MD adherence and dementia risk was increased in individuals with lower educational attainment.

Berti et al., [108], performed a longitudinal cohort study with the aim to examine the effects of higher vs. lower MD adherence on changes in AD biomarkers over a 3-year period in cognitively normal, middle-aged adults. In the baseline comparisons, MD+ group showed improved glucose metabolism and lower amyloid deposition in AD-affected brain regions compared to the MeDi− group. Over the follow-up period, the MD− group displayed declining glucose metabolism (CMRglc) and increased amyloid deposition in AD-related brain regions. Furthermore, the MD+ group showed less decline in CMRglc and less increase in amyloid deposition over time, suggesting protective effects of higher MD adherence against AD pathology. However, no significant effects were observed on brain volume or atrophy, signifying that the effects of diet on structural brain changes may entail a longer follow-up period. The study indicates that higher MD adherence was associated with 1.5 to 3.5 years of protection against a decrease in brain volume and atrophy.

Klinedinst et al. [34] explored the impact of specific dietary components on cognitive outcomes in individuals with genetic risk factors, showing that daily cheese intake and red wine consumption were associated with better cognitive outcomes in those with a family history of dementia. This suggests that certain components of the MD may be particularly beneficial for individuals at genetic risk of cognitive decline.

The cross-sectional study by Karstens et al. [101] explored the impact of the MD on brain structure, finding that higher adherence was associated with larger dentate gyrus volumes and better performance in learning and memory tasks. This neuroimaging evidence supports the role of diet in preserving brain health and cognitive function.

McGrattan (2021) conducted a pilot RCT showing that an MD intervention could improve cognitive performance in individuals with MCI and SCD. The study found that community-based recruitment was particularly effective in engaging participants, highlighting the importance of accessibility and support in dietary interventions.

A previous study by Haring et al., 2016 [32] investing the dietary patterns of aMED, HEI-2010, AHEI-2010, DASH in older postmenopausal women who were followed up after 9 years, some of study participants developed either MCI or probable dementia (PD). However, no significant relations were identified across the quintiles of aMED, HEI-2-2-, AHEI-2020 and DASH adherence.

A study by Olsson et al. 2015 [47] investigated in elderly Swedish men, how the Modified MD Score (mMDS) and low carbohydrate-high protein diet (LCHP) are related to the incidence of cognitive decline and dysfunction. However, the study found no association between mMDS and LCHP and cognitive decline.

The effect of MD and WD on AD biomarkers, cerebral perfusion and cognition was investigated by, Hoscheidt et al. [131] via a RCT that consisted of normal cognitive and MCI individuals. In the dietary group, Aβ40 concentration was decrease with the MD but increased with WD. No changes were reported for MCI. The NC group showed increased ratios following MD and reduced ratios after WD but the MCI group showed the reverse pattern. Significant cerebral perfusion was observed in the left inferior frontal cortex, right middle temporal gyrus and para-hippocampal gyri for MD >WD, while a Western diet had the opposite effect.

Corley et al., 2020 [50], investigating dietary patterns and cognition in dementia free subjects over a 12-year period. The Higher MD adherence at baseline associated with better verbal ability. Higher adherence to traditional diet associated with lower verbal ability. MD associated with steeper decline in verbal ability over 12 years. Higher MD adherence at baseline associated with better verbal ability. Moreover, higher adherence to traditional diet associated with lower verbal ability. However, a

healthier baseline diet was unable to predict a decreased risk of either global or domain specific cognitive decline.

Lastly, Tzekaki et al. 2019 [43], investigated the administration of EVOO in MCI patients as a potential therapy to prevent AD progress. MCI individuals treated with EVOO had decreased plasminogen activator inhibitor (PAI-1) and a2-antiplasmin serum concentrations compared to control group. Additionally, the MCI group that received one-year EVOO demonstrated a Aβ1-42/ Aβ1-40 ratio comparable to that of healthy individuals. The MCI+EVOO group also displayed, a decreased concentration of tau protein concentration and Malondialdehyde (MDA) oxidative stress marker in comparison to the other groups. Therefore, EVOO therapy may prevent progression of MCI to AD by decreasing fibrinolytic factors (PAI-1 and a2-antiplasmin), tau and Aβ amyloid and MDA.

Genetic Considerations play a role in how diet affects cognitive health, particularly concerning the APOE genotype. Various studies have highlighted the association between genetic predisposition and diet.

Dhana et al. [77,98] highlighted that adherence to multiple healthy lifestyle factors, including the MIND diet, was associated with a significantly reduced risk of developing Alzheimer's dementia and improved mortality rates among those with higher adherence. Vu et al. [77] showed that higher adherence to the MIND diet was associated with lower dementia risk, independently of genetic predisposition, indicating the broad applicability of dietary interventions across different genetic backgrounds.

Nordic Diet

The ND is similar to the MD, where it emphasizes whole, minimally processed foods native to the Nordic countries (Sweden, Denmark, Norway and Finland), it includes the high consumption of berries, vegetables, whole grains, fatty fish, legumes, and rapeseed oil. The ND may also have protective effects on neuronal cells and by preventing cognitive decline and promoting cognitive health. Berries rich in antioxidants, fatty fish with Omega-3 fatty acids, and whole grains with low glycemic indices help reduce oxidative stress, inflammation, and improve cardiovascular health, which are all linked to lower risks of dementia and AD.

A previous study by, Shakersain et al. [30] investigated the role of Nordic Prudent Dietary Pattern (NPDP) adherence in the Swedish population. The study revealed that found that moderate to high adherence to the NPDPwas associated with a decrease in cognitive decline and a lower risk of significant cognitive decline based on the MMSE score, this association was stronger with moderate to intense, physical mental of social related activities. Another study by Shakersain et al. [29], that observed that individuals with high adherence to all dietary patterns had the highest level of education, were physically very active, and a lower proportion were current smokers.

Wu et al. [31] investigated how the NPDP may prolong survival with good mental physical functioning among older adults. During the follow-up period, 1,074 participants survived with no dementia developed. The study indicated that high NPDP adherence was associated with a 20% higher probability of dementia-free and disability-free survival, prolonging lifespan without mental and physical disability by 1.24 years. The above-mentioned study supports the notion that high NPDP adherence and maintain lifestyle behaviours such physical activity, mental and social activities support healthy aging and may likely prevent AD, dementia and MCI.
